# Pycnogonida collection of the Shirshov Institute of Oceanology, Russian Academy of Sciences

**DOI:** 10.3897/BDJ.13.e142496

**Published:** 2025-04-01

**Authors:** Zoya Dudnik, Antonina Kremenetskaia

**Affiliations:** 1 Shirshov Institute of Oceanology, Russian Academy of Sciences, Moscow, Russia Shirshov Institute of Oceanology, Russian Academy of Sciences Moscow Russia

**Keywords:** marine fauna, Pantopoda, sea spiders, zoological collections

## Abstract

**Background:**

This dataset comprises detailed information on 2,134 Pycnogonida specimens stored in the Ocean Benthic Fauna collection (collection code: OBFc) of the Shirshov Institute of Oceanology (IORAS). These specimens were collected over a span of 77 years, since 1947, from 996 distinct localities across various regions of the World Ocean.

The IORAS Pycnogonida collection stands out for its wide range of Pycnogonida species, including both common and exceptionally rare specimens, some of which are unique to this collection. This diversity makes the dataset an invaluable resource for taxonomists, ecologists and marine biologists, particularly those studying sea spiders. By providing comprehensive data on species distribution and diversity, the collection plays a key role in advancing our understanding of these intriguing marine arthropods. It serves as a vital reference for scientific research, aiding in species identification, the study of ecological relationships and the analysis of biogeographic patterns worldwide.

**New information:**

The whole Pycnogonida collection of the Shirshov Institute of Oceanology has undergone a meticulous revision and digitisation process to compile a comprehensive dataset on the geographic, bathymetric and taxonomic specimen distribution. This effort includes the documentation and imaging of the type collection as well as of rare and unique records. The resulting dataset serves as a valuable resource for a variety of scientific disciplines, including taxonomic studies, biodiversity research and biogeographic analyses. This dataset contributes to our understanding of marine biodiversity and the distribution patterns of Pycnogonida across different oceanic regions and depths.

## Introduction

Pycnogonida (Gr. pyknos, meaning "thick" or "dense" + Gr. gony, meaning "knee"), commonly known as sea spiders, represent an ancient class of arthropods, sister to the remaining Chelicerata (Dunlop and Arango 2005). Sea spiders exhibit a remarkable range in size, with leg spans varying from just a few millimetres to over 75 centimetres, particularly in polar regions where they tend to grow larger (Arnaud and Bamber 1987, Bamber 2007). To date, over 1,300 species of extant pycnogonids have been described (Bamber et al. 2024).

Sea spiders are free-living marine invertebrates, distributed from the Poles to the Equator and from the littoral to the hadal depths. Highly adaptable, they inhabit nearly every type of marine environment, from the most biodiverse coral reefs to the cold, oxygen-rich waters of polar regions; from coastal seagrass beds to the stark, nutrient-poor environments of the deep sea; from rocky shore communities to soft sediment habitats, such as mudflats or sandy sea floors (Arnaud and Bamber 1987). Some species of sea spiders inhabit hydrothermal zones (Turpaeva 1988). Although pycnogonids are primarily benthic animals, they possess the ability to rise into the water column (Morgan 1972, Clark and Carpenter 1977). They are most commonly collected using bottom trawls, dredges (Gordon 1932) and occasionally benthic traps (Child 1998), though they are also rarely found in plankton nets (Ohshima 1933) and other pelagic fishing gear.

Sea spiders feed by using a long proboscis to suck nutrients from soft-bodied invertebrates. Adult sea spiders are mostly carnivorous predators or, rarely, parasites feeding on the soft tissues of hydroids, actinians, sponges, bryozoans, corals and molluscs (Dietz et al. 2018).

Sea spiders have a unique body structure where their organs, including parts of the digestive and reproductive systems, extend into their legs. Pycnogonids typically have four pairs of long legs, though some species may have five or six pairs (Hedgpeth 1947), all attached to a comparatively small body. The trunk is divided into segments. The first segment, the cephalosoma, features the proboscis, the ocular tubercle with four eyes, three pairs of appendages – the chelifores, the palps and the ovigers and the first pair of walking legs. Behind the cephalosoma, there are 3-5 trunk segments, each bearing a pair of walking legs. The last trunk segment also carries the abdomen, ending in the anal orifice (Arnaud and Bamber 1987).

The ovigerous legs of pycnogonids play a crucial role in reproduction, as males (except those belonging to the Colossendeidae family (Brenneis and Wagner 2023) use these specialised limbs to carry their offspring. Nearly all pycnogonids are dioecious. After the female lays her eggs, she transfers them to the male, who then fertilises them. The male either forms cocoons around the fertilised eggs on his ovigerous legs or immerses his legs in a shapeless mass of eggs. The eggs in the cocoons are held together by a gelatinous substance secreted by cement glands located on the femoral segments of the male's walking legs. The male continues to carry the cocoons until the very latest stages of embryonic development, often until hatching and sometimes even until the larvae are fully developed (Bain and Govedich 2004). The larvae themselves are highly diverse in both size and lifestyle (Bogomolova and Malakhov 2006, Brenneis et al. 2017).

The largest and most significant scientific collections of Pycnogonida specimens are preserved in major natural history museums and research institutions worldwide. Some of the most notable collections are in the Natural History Museum (NHM, London, UK), Smithsonian National Museum of Natural History (NMNH, collection code USNM, Washington, D.C., USA), Muséum national d'Histoire naturelle (MNHN, collection code IU, Paris, France), Natural History Museum Denmark (NHMD, Copenhagen, Denmark), Australian Museum (AM, Sydney, Australia) and South African Museum (SAM, Cape Town, South Africa).

### The IORAS Pycnogonida collection

The IORAS Pycnogonida collection was primarily identified and curated by Elena Petrovna Turpaeva (1923–2017), a prominent Soviet and Russian zoologist and an expert on sea spider taxonomy and marine fouling. Elena Petrovna worked in the IORAS since 1950 and described two new genera of Pycnogonida - *Hedgpethia* Turpaeva, 1973 (Turpaeva 1973) and *Anisopes* Turpaeva, 1998 (Turpaeva 1998) (currently accepted as *Sericosura* Fry & Hedgpeth, 1969), 60 new species and 11 new subspecies. The collection houses 110 type specimens representing 51 species and 11 subspecies (Table 1). A significant part of the data on pycnogonids in the collection was published in more than 30 works by E.P. Turpaeva and A.K. Rajsky.

The entire IORAS Pycnogonida collection was digitised using Specify 6 software (Specify Collections Consortium 2023). The catalogue includes scientific names in accordance with the World Register of Marine Species (WoRMS) (WoRMS Editorial Board 2024), along with collection date, coordinates, depth, images and other data for each collection lot. Subsequently, this catalogue was exported to a Darwin Core occurrence dataset and made accessible via the Global Biodiversity Information Facility (GBIF) (GBIF.org 2024b).

## Sampling methods

### Sampling description

The collection specimens were mainly obtained during the research cruises using Sigsbee trawls (761 specimens), bottom trawls (176 specimens), Agassiz trawls (155 specimens), "Okean" grabs 0.25 (98 specimens) and dredges (97 specimens). In total, 1,472 pycnogonid specimens were obtained by different trawl types. The most diverse catches, in terms of species number, were obtained in the Weddell Sea, Bransfield Strait and North-West Pacific Ocean (Table 2).

## Geographic coverage

### Description

**Figure 11.**
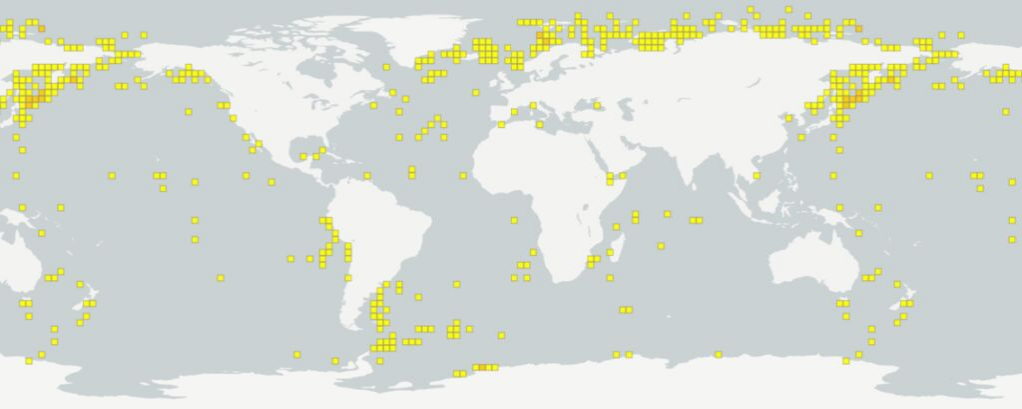
Sampling localities of IORAS pycnogonids (GBIF.org 2024a).

**Figure 12.**
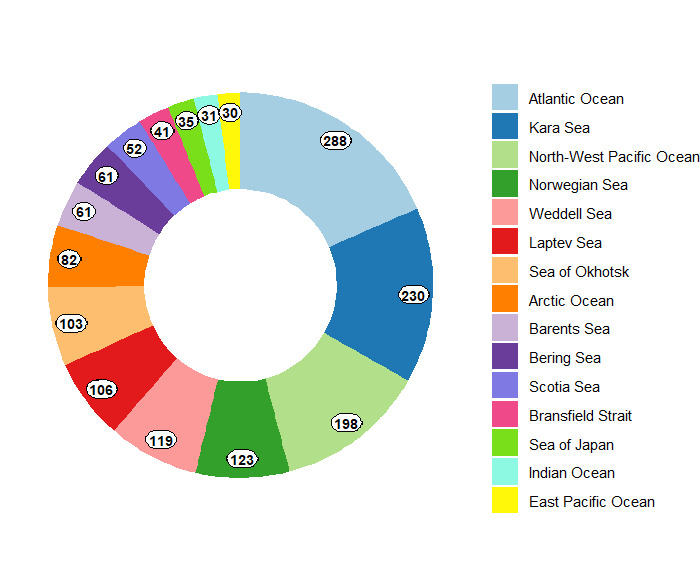
Geographic origin of pycnogonids deposited in the IORAS collection. Numbers correspond to the amount of collection lots.

The collection encompasses samples collected since 1947 through 144 research cruises at 996 stations (localities) in different areas of the World Ocean (Fig. 11). It is notably robust in specimens from the polar regions, including both the Arctic and Antarctic and the North-West Pacific regions. Most of the specimens were obtained from the Soviet and Russian cruises onboard RV (research vessel) Vityaz, Akademik M. Keldysh and Dmitry Mendeleev, focusing on the Barents, Kara, Bering Sea and the Sea of Okhotsk. Additionally, numerous samples were obtained from RV Polarstern research cruises in the Bransfield Strait, Drake Passage, Laptev Sea and Weddell Sea (Fig. 12).

**Figure 13.**
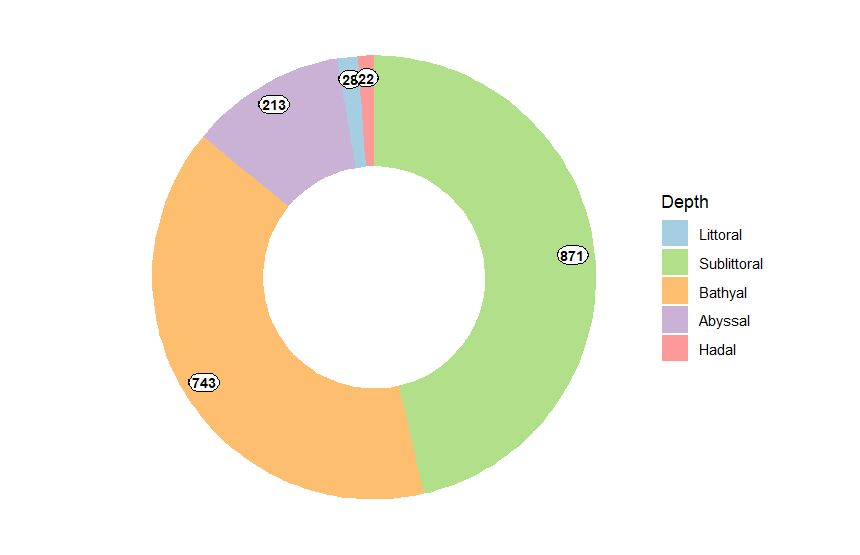
Bathymetric distribution of IORAS pycnogonids. Numbers correspond to the amount of collection lots.

**Figure 14.**
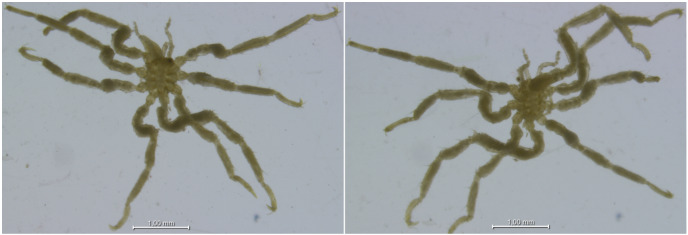
*Achelia* sp. from the Mariana Trench collected at 10,700 m (cat. INV0001448).

**Figure 15.**
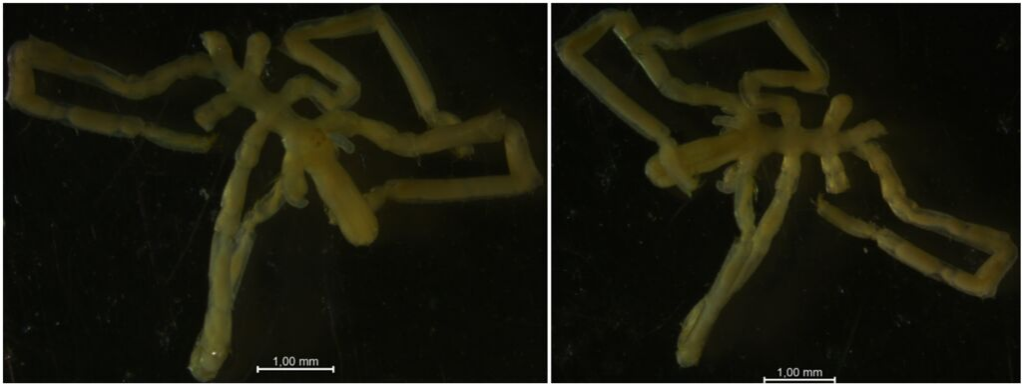
*Endeis* sp. from the Mariana Trench collected at 10700 m (cat. INV0005251).

The specimens were collected from all depth ranges (Fig. 13). The collection includes numerous deep-sea representatives, with 743 lots from bathyal depths and 213 lots from abyssal depths. Additionally, it features 19 specimens from the ultra-abyssal (hadal) zone of the Kurile-Kamchatka, Japan, Mariana, Peru, South Sandwich, Volkano, Izu-Bonin and Aleutian trenches (Table 3). Amongst these, the deepest records are *Achelia* sp. (catalogue number INV0001448) and *Endeis* sp. (INV0005251), both collected from the Mariana Trench at 10,700 m (Figs 14, 15).

In addition to deep-sea samples, the collection includes 899 littoral and sublittoral specimens. A substantial portion of this collection was obtained from the littoral of the Kola Peninsula (including the MSU (Moscow State University) White Sea biological station area), Primorskii Kray, Commander and Aleutian Islands and Novaya Zemlya Archipelago.

## Taxonomic coverage

### Description

**Figure 16.**
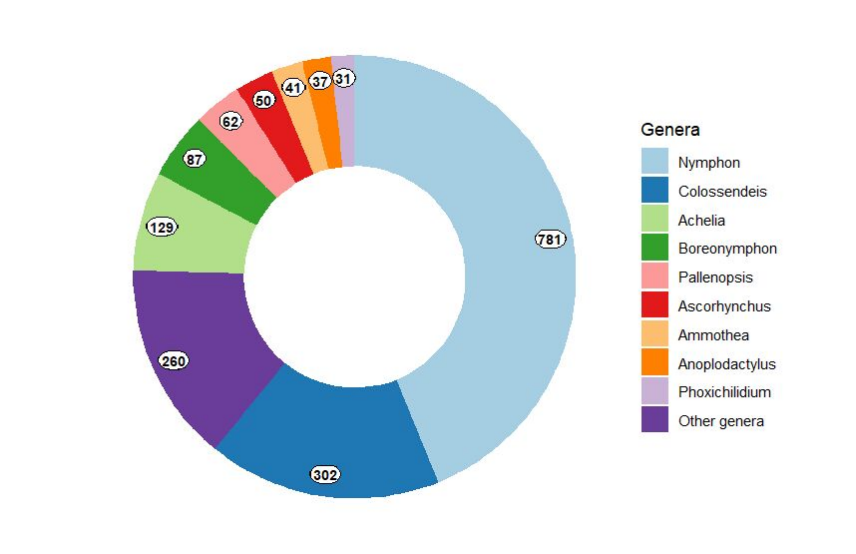
Most represented genera by number of lots (shown in numbers) in the IORAS collection.

A total of 74% of the collection specimens are identified to the species level. The collection includes 291 species belonging to 46 genera and 12 families of Pycnogonida (Fig. 16, Table 4).

**Figure 17.**
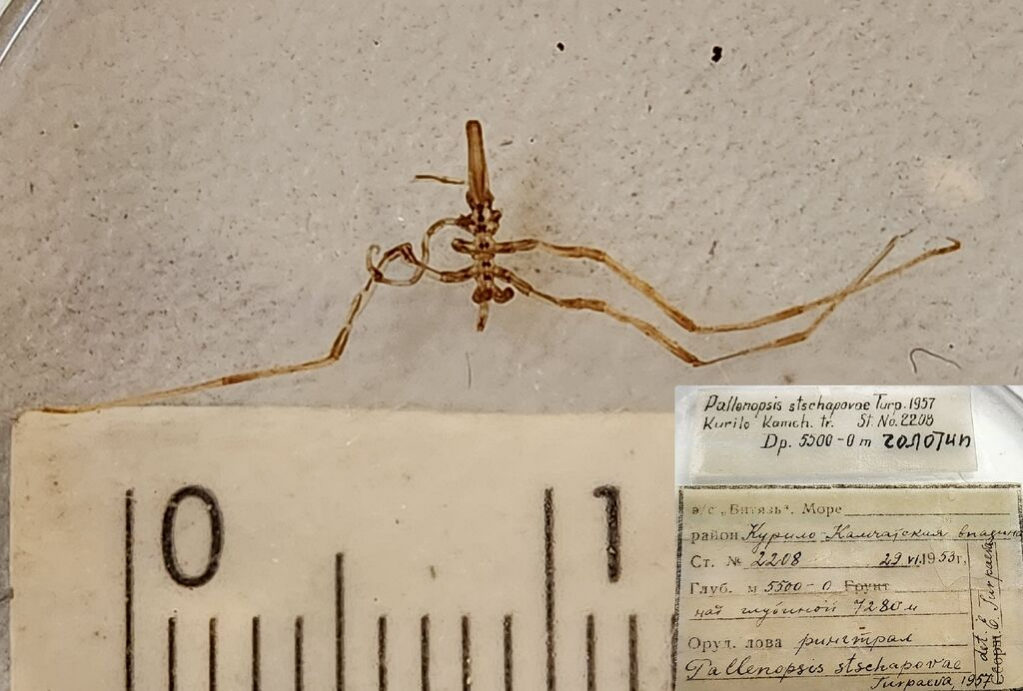
*Pallenopsisstschapovae* (holotype, cat. INV0001062).

In addition, the collection includes rare pelagic sea spider *Pallenopsisstschapovae* (Turpaeva 1957) (holotype, catalogue number INV0001062) collected on 22-06-1953 by the ring trawl during RV Vityaz 14^th^ cruise (Fig. 17). This species is currently accepted under *Bathypallenopsistritonis* (Hoek, 1883) (Hoek 1883) according to Child (1995) and Bamber (2002).

**Figure 18.**
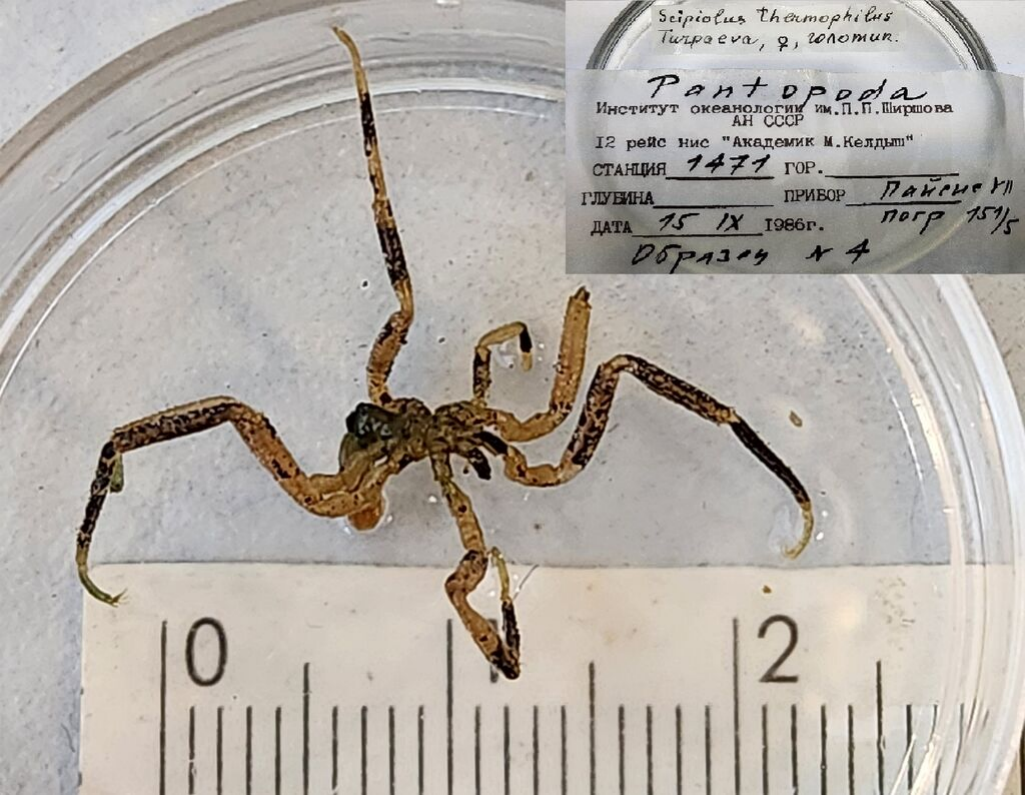
*Scipiolusthermophilus* (holotype, cat. INV0000925).

**Figure 19.**
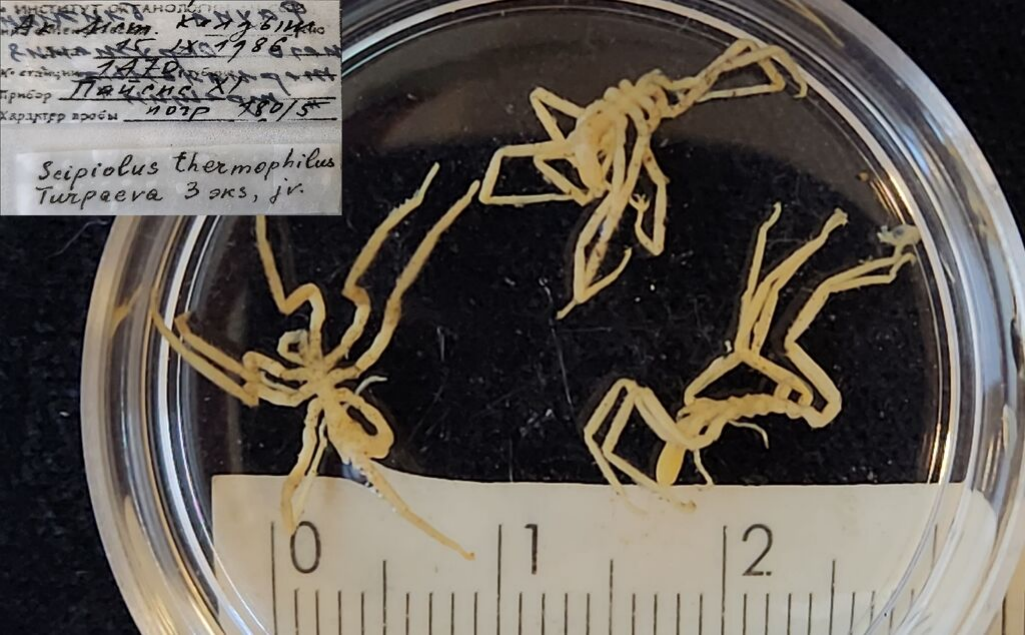
*Scipiolusthermophilus* (cat. INV0000926).

The IORAS collection also includes specimens of the hydrothermal pycnogonid *Scipiolusthermophilus* (Turpaeva 1988) which is currently accepted as *Sericosuraverenae* (Child, 1987) according to Child (1987) and Bamber (2009). The samples were collected on 15/09/1986 using the deep manned submersibles Pisces VII and XI at a depth of 1,800 m in the hydrothermal vent of the Juan de Fuca Ridge during the 12^th^ cruise of RV Akademik Mstislav Keldysh (holotype from station 1471, catalogue number INV0000925 (Fig. 18) and three specimens from station 1470, catalogue number INV0000926 (Fig. 19). The holotype specimen (Fig. 19) is covered with a black crust-like coating on the surface of the body and limbs. The white coating on the three specimens shown in Fig. 20 is formed by bacterial threads located on the cuticle of the distal parts of the limbs, in between the setae. White lumps, likely bacterial mats, also cover the distal parts of the walking legs, especially the claws and accessory claws of these specimens.

### Taxa included

**Table:** 

Rank	Scientific Name	
family	Nymphonidae	
family	Ammotheidae	
family	Austrodecidae	
family	Ascorhynchidae	
family	Colossendeidae	
family	Callipallenidae	
family	Pycnogonidae	
family	Phoxichilidiidae	
family	Endeidae	
family	Pallenopsidae	
family	Rhynchothoracidae	

## Temporal coverage

**Data range:** 1947-7-15 – 2022-2-08.

## Collection data

### Collection name

Ocean Benthic Fauna collection

### Collection identifier

OBFc

### Specimen preservation method

Alcohol

### Curatorial unit

Laboratory of Ocean Benthic Fauna

## Usage licence

### Usage licence

Other

### IP rights notes

Creative Commons Attribution Non-Commercial (CC-BY-NC) 4.0 Licence

## Data resources

### Data package title

Pycnogonida collection of the Shirshov Institute of Oceanology, Laboratory of Ocean Benthic Fauna

### Resource link

https://doi.org/10.15468/azchd4

### Alternative identifiers

https://gbif.ocean.ru/ipt/resource?r=pycnogonida_ioras

### Number of data sets

1

### Data set 1.

#### Data set name

Pycnogonida collection of the Shirshov Institute of Oceanology, Laboratory of Ocean Benthic Fauna

#### Data format

Darwin Core Archive

#### Character set

UTF-8

#### Download URL

https://gbif.ocean.ru/ipt/archive.do?r=pycnogonida_ioras&v=1.43

#### Data format version

18-09-2023

#### Description

The dataset contains data on the Pycnogonida specimens stored in the Ocean Benthic Fauna collection of the Shirshov Institute of Oceanology (IORAS).

**Data set 1.** 

Column label	Column description
occurrenceID	An identifier for the dwc:Occurrence (as opposed to a particular digital record of the dwc:Occurrence).
institutionID	An identifier for the institution having custody of the object(s) or information referred to in the record.
collectionID	An identifier for the collection or dataset from which the record was derived.
institutionCode	The name (or acronym) in use by the institution having custody of the object(s) or information referred to in the record.
collectionCode	The name, acronym, coden or initialism identifying the collection or dataset from which the record was derived.
ownerInstitutionCode	The name (or acronym) in use by the institution having ownership of the object(s) or information referred to in the record.
basisOfRecord	The specific nature of the data record.
catalogNumber	An identifier for the record within the dataset or collection.
eventRemarks	Name of RV (research vessel) on board which the original dwc:Occurrence recording was made.
individualCount	The number of individuals present at the time of the dwc:Occurrence.
occurrenceStatus	A statement about the presence or absence of a dwc:Taxon at a dcterms:Location.
preparations	A preparation or preservation method for a specimen.
associatedMedia	A list of URLs of media associated with the dwc:Occurrence.
eventID	An identifier for the set of information associated with a dwc:Event in the format "RV name_cruise number_st.number".
parentEventID	An identifier for the cruise number where the original dwc:Occurrence was recorded.
fieldNumber	An identifier for the station number where the original dwc:Occurrence was recorded.
eventDate	The date when the dwc:Event was recorded.
samplingProtocol	The names of the methods or protocols used during a dwc:Event. Such as trawls (Sigsbee, Agassiz, Galathea etc.), dredges, grabs (common and television-guided TV grabs), box corers (common and television-guided TV multicorers), submersibles (HOVs (human occupied vehicles) Mir-1, Mir-2 and Pisces and ROVs (remotely operated vehicles).
waterBody	The name of the water body in which the dcterms:Location occurs.
islandGroup	The name of the island group in which the dcterms:Location occurs.
country	The name of the country or major administrative unit in which the dcterms:Location occurs.
countryCode	The standard code for the country in which the dcterms:Location occurs.
locality	The original textual description of the place.
verbatimDepth	The original description of the depth below the local surface. Range means depths of start and end of sampling.
decimalLatitude	The geographic latitude (in decimal degrees, using the spatial reference system given in dwc:geodeticDatum) of the geographic centre of a dcterms:Location. Positive values are north of the Equator, negative values are south of it.
decimalLongitude	The geographic longitude (in decimal degrees, using the spatial reference system given in dwc:geodeticDatum) of the geographic centre of a dcterms:Location. Positive values are east of the Greenwich Meridian, negative values are west of it.
geodeticDatum	The ellipsoid, geodetic datum or spatial reference system (SRS), upon which the geographic coordinates given in dwc:decimalLatitude and dwc:decimalLongitude are based.
verbatimIdentification	A string representing the taxonomic identification as it appeared in the original record.
typeStatus	Nomenclatural type (type status) applied to the subject.
identifiedBy	Person name who assigned the dwc:Taxon to the subject.
scientificName	The name in lowest level taxonomic rank that can be determined.
nameAccordingTo	The reference to the source in which the specific taxon concept circumscription is defined or implied.
kingdom	The full scientific name of the kingdom in which the dwc:Taxon is classified.
phylum	The full scientific name of the phylum or division in which the dwc:Taxon is classified.
class	The full scientific name of the class in which the dwc:Taxon is classified.
order	The full scientific name of the order in which the dwc:Taxon is classified.
family	The full scientific name of the subfamily in which the dwc:Taxon is classified.
taxonRank	The taxonomic rank of the most specific name in the dwc:scientificName.

## Figures and Tables

**Figure 1a.**
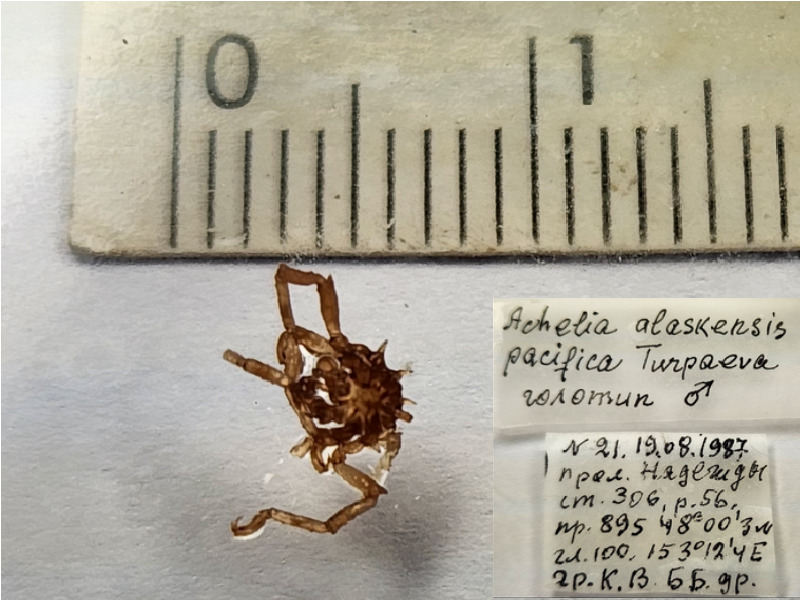
*Acheliaalaskensispacifica* (cat. INV0001682);

**Figure 1b.**
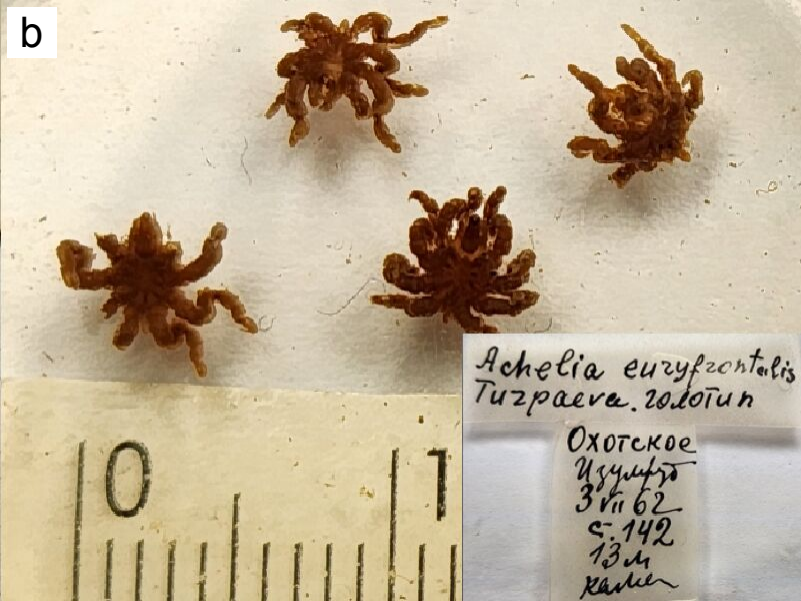
*Acheliaeuryfrontalis* (cat. INV0000971) (the holotype is stated as juvenile male specimen, a separate taxonomic revision is needed to isolate it);

**Figure 1c.**
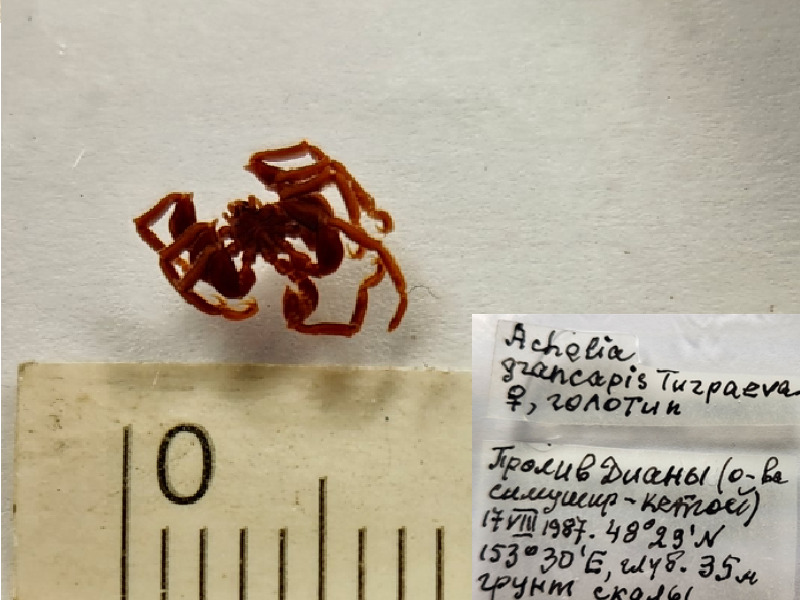
*Acheliagrancapis* (cat. INV0001690);

**Figure 1d.**
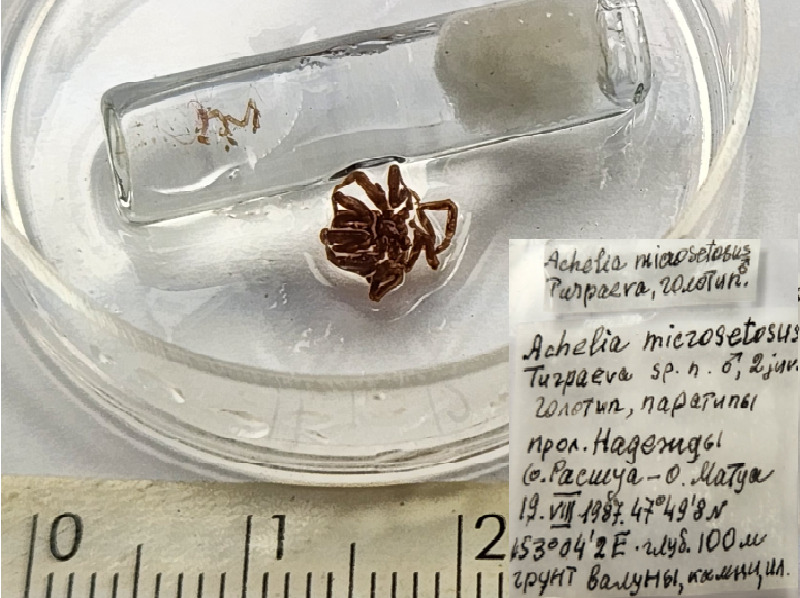
*Acheliamicrosetosa* (cat. INV0001688);

**Figure 1e.**
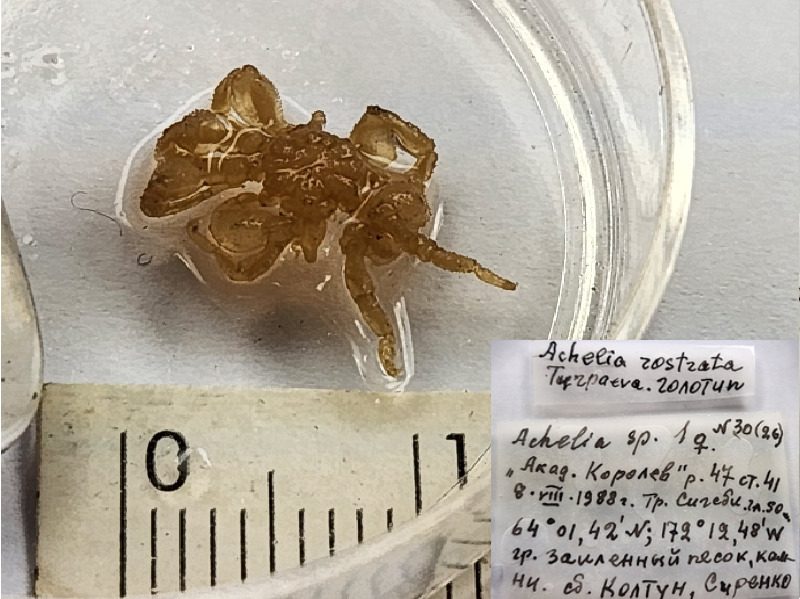
*Acheliarostrata* (cat. INV0000968);

**Figure 1f.**
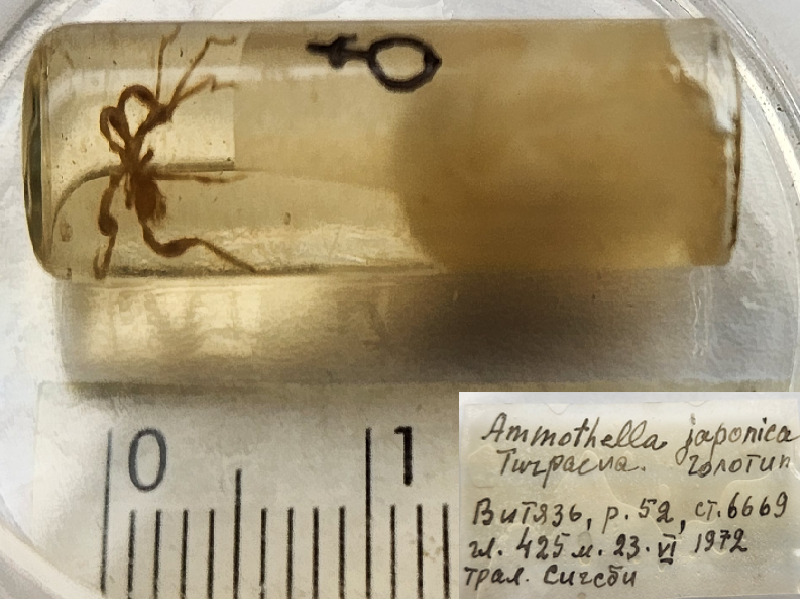
*Ammothellajaponica* (cat. INV0000928).

**Figure 2a.**
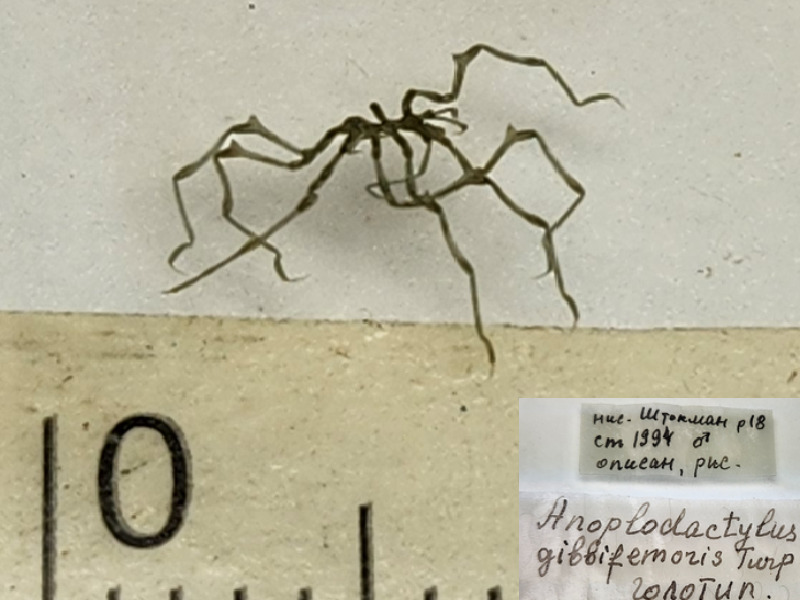
*Anoplodactylusgibbifemoris* (cat. INV0002357);

**Figure 2b.**
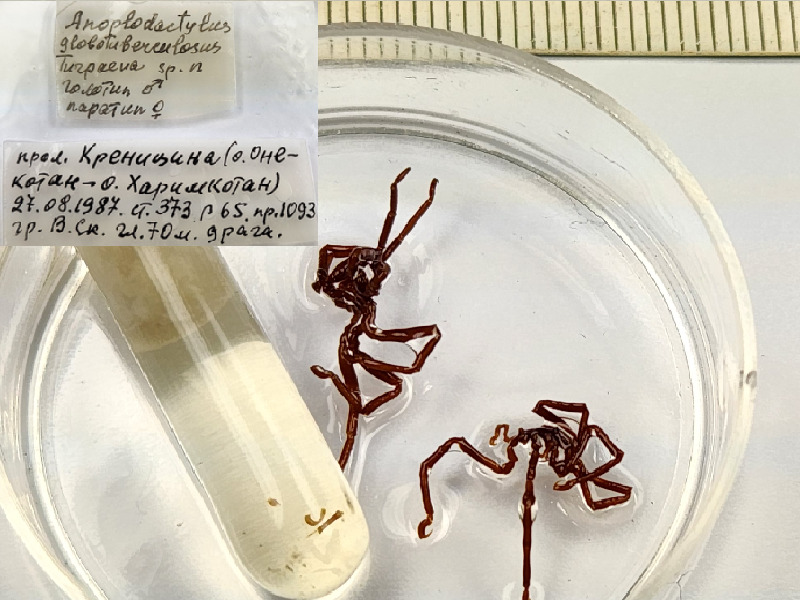
*Anoplodactylusglobotuberculosus* (cat. INV0001244) (the holotype (male) and the paratype (female) in one vial, further morphological study is required to differentiate them);

**Figure 2c.**
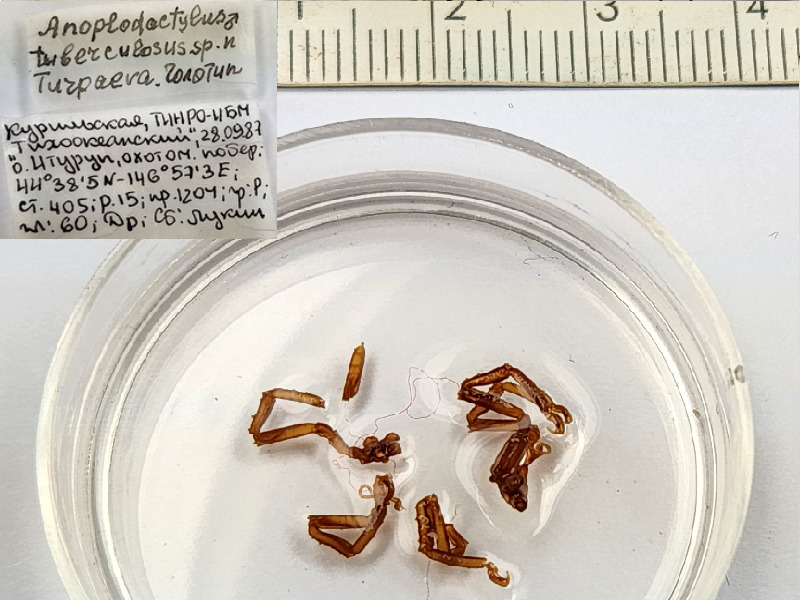
*Anoplodactylustuberculosus* (cat. INV0001245);

**Figure 2d.**
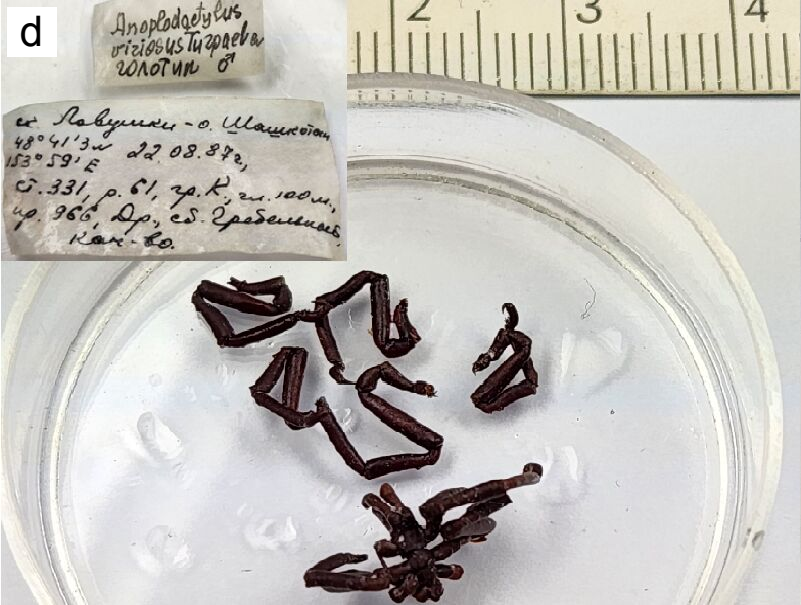
*Anoplodactylusviriosus* (cat. INV0001250);

**Figure 2e.**
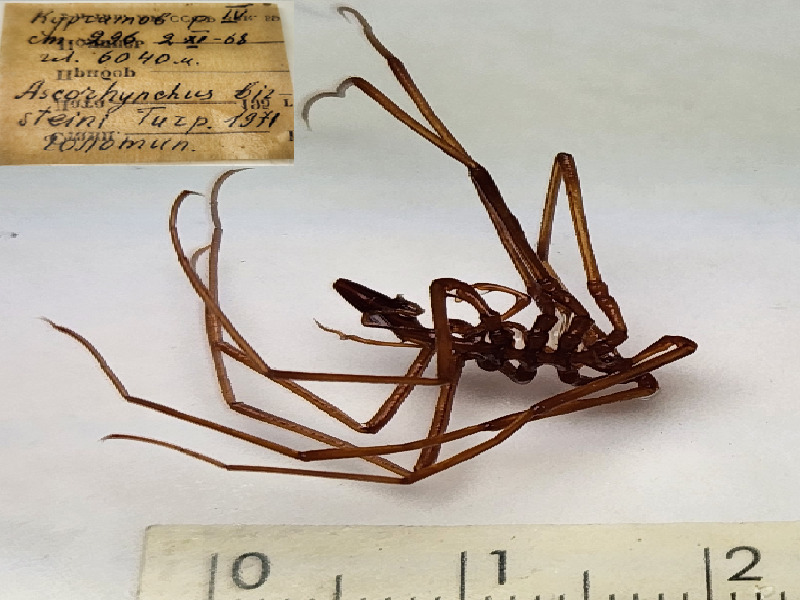
*Ascorhynchusbirsteini* (cat. INV0002362);

**Figure 2f.**
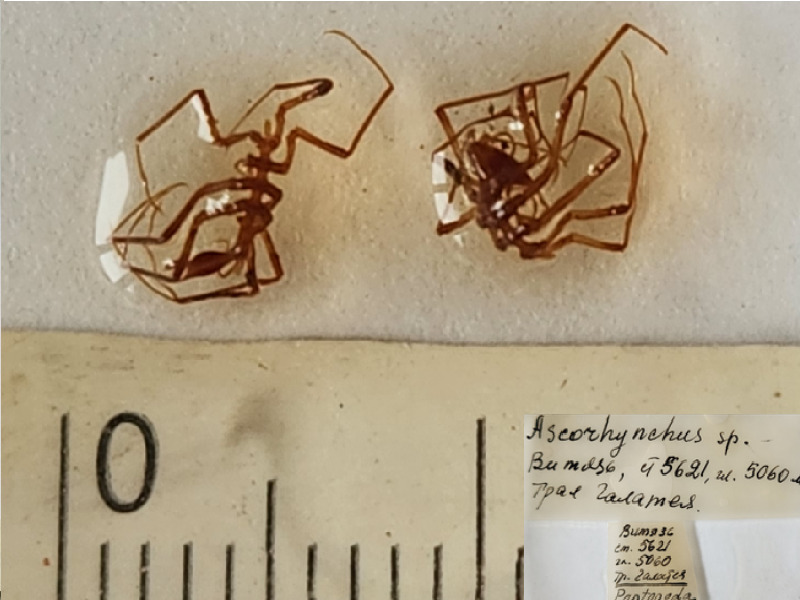
*Ascorhynchusbucerus* (cat. INV0000990) (2 syntypes, no holotype designated).

**Figure 3a.**
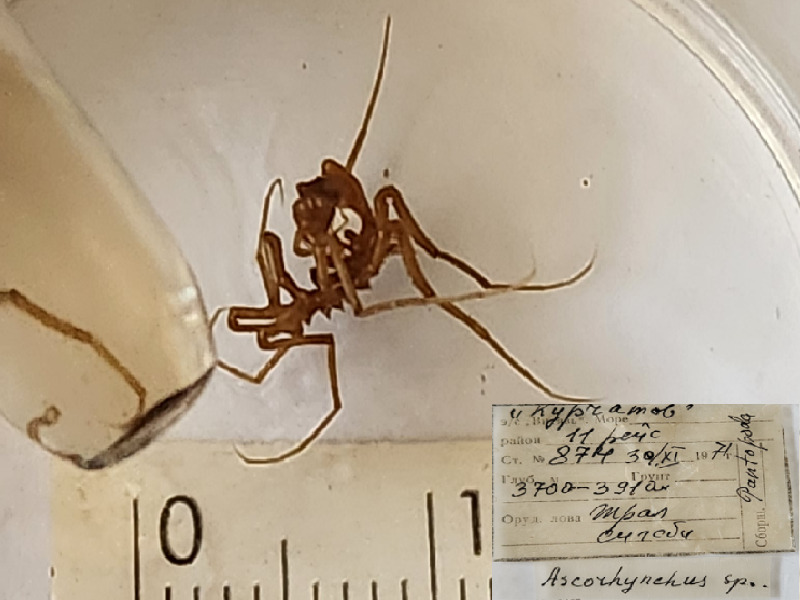
*Ascorhynchushedgpethi* (cat. INV0000965);

**Figure 3b.**
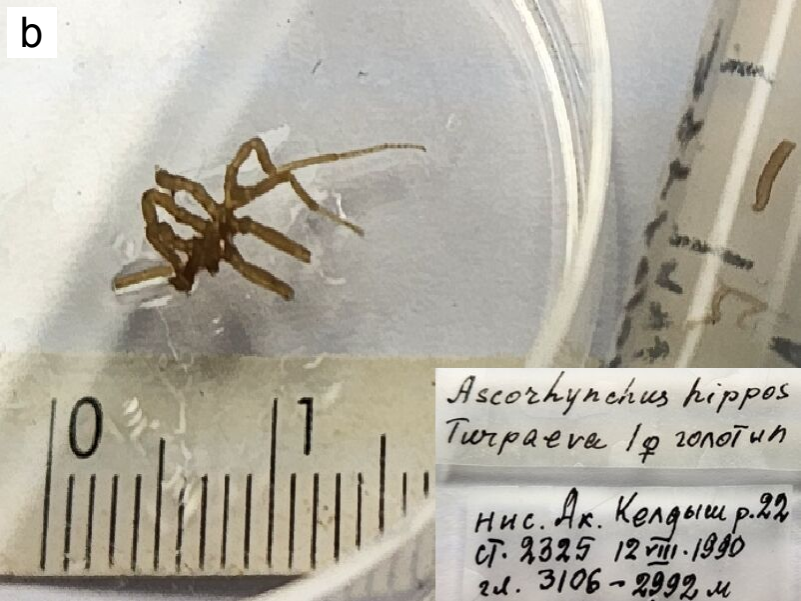
*Ascorhynchushippos* (cat. INV0000793);

**Figure 3c.**
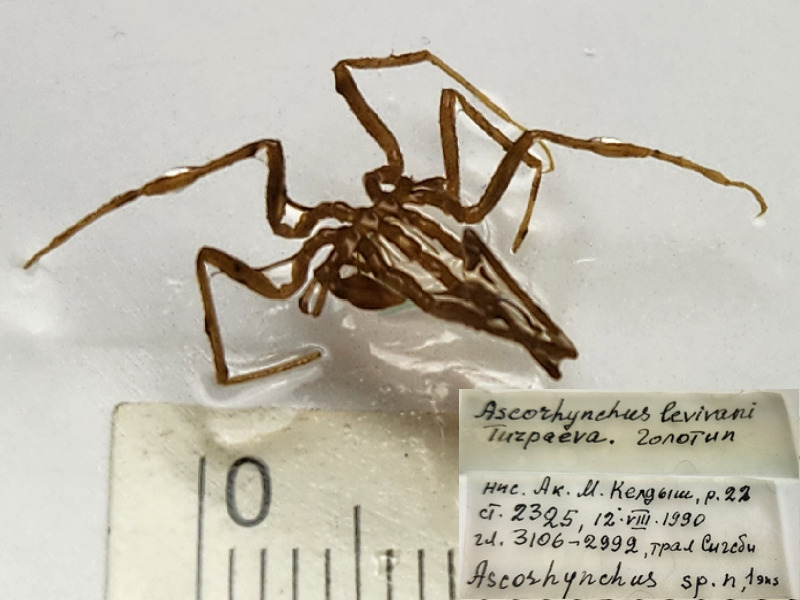
*Ascorhynchuslevivani* (cat. INV0002340);

**Figure 3d.**
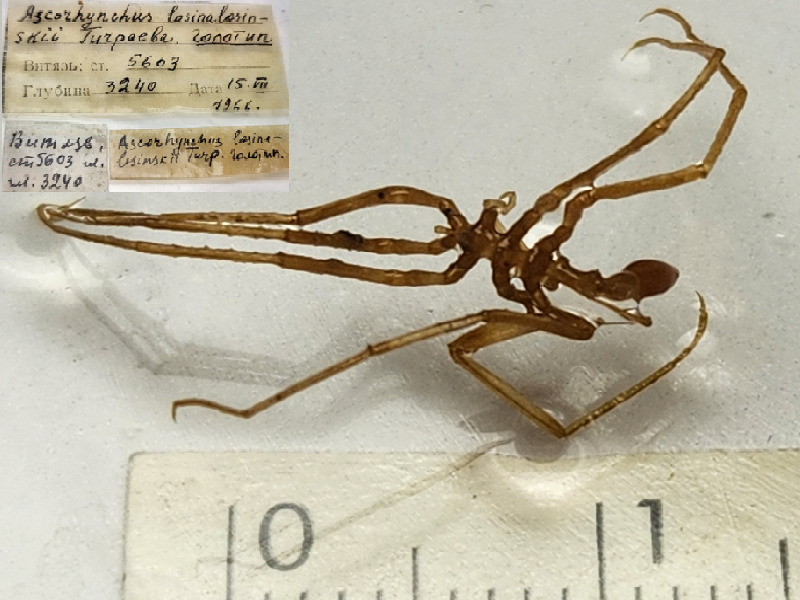
*Ascorhynchuslosinalosinskii* (cat. INV0002361);

**Figure 3e.**
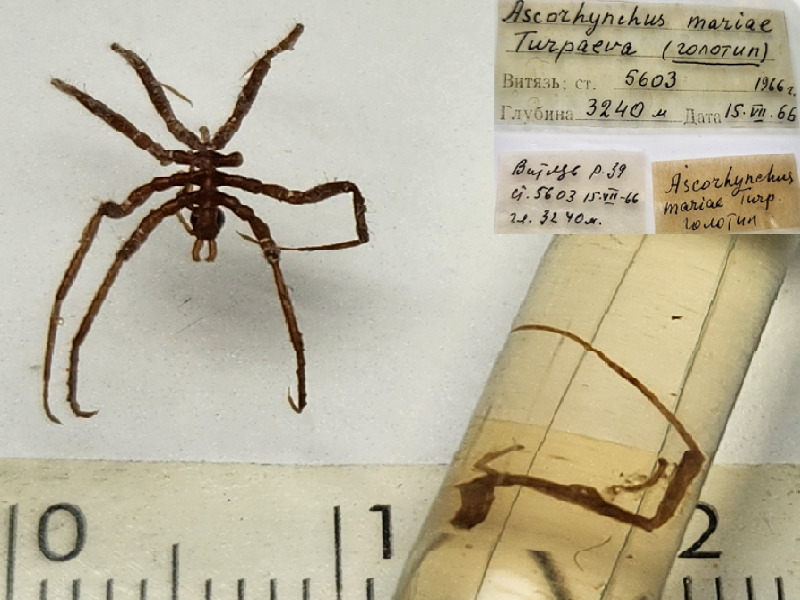
*Ascorhynchusmariae* (cat. INV0002353);

**Figure 3f.**
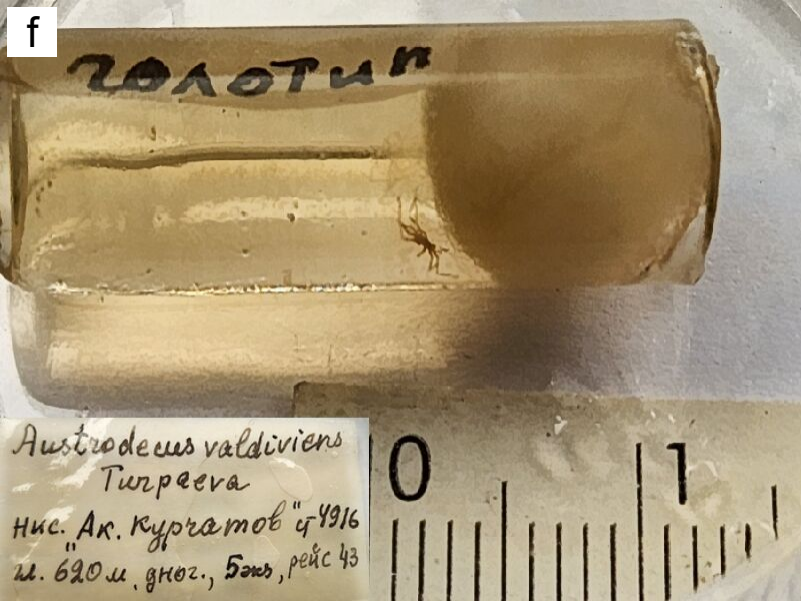
*Austrodecusvaldiviens* (cat. INV0000927).

**Figure 4a.**
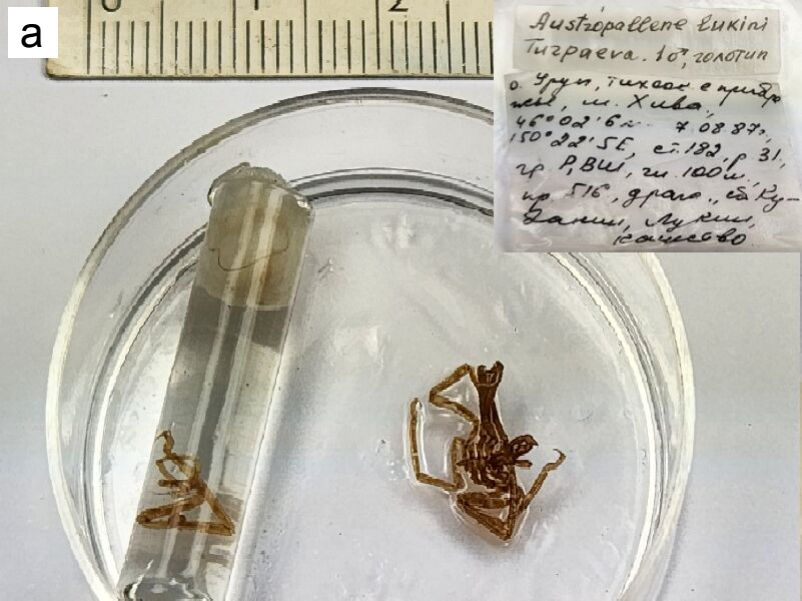
*Austropallenelukini* (cat. INV0001228);

**Figure 4b.**
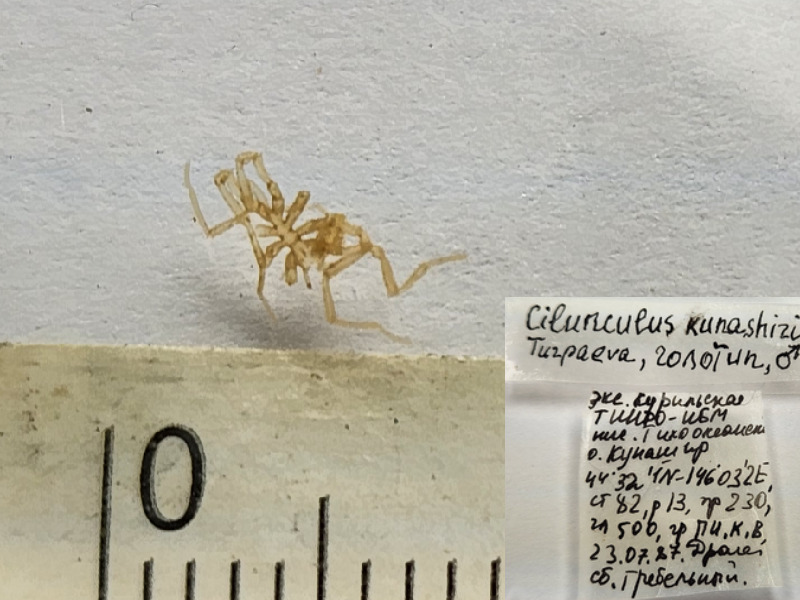
*Cilunculuskunashiri* (cat. INV0001757);

**Figure 4c.**
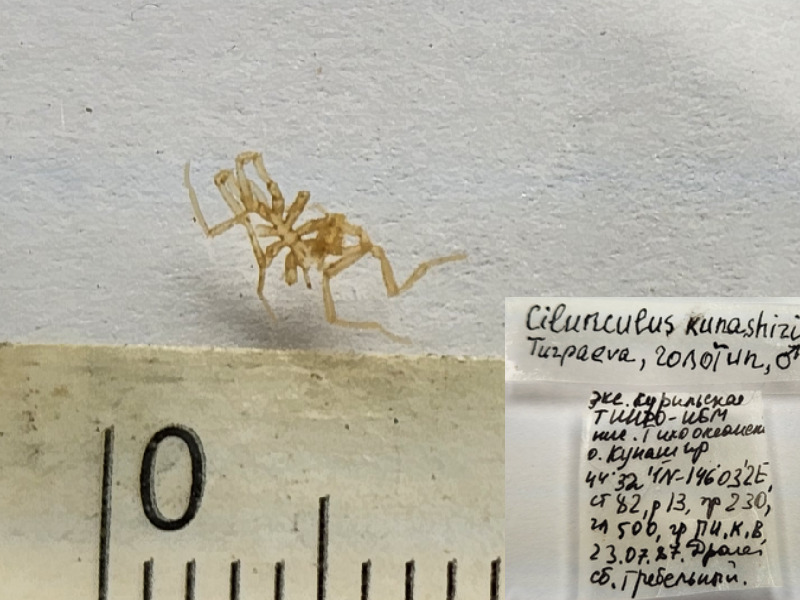
*Cilunculusmisesetosus* (cat. INV0000969);

**Figure 4d.**
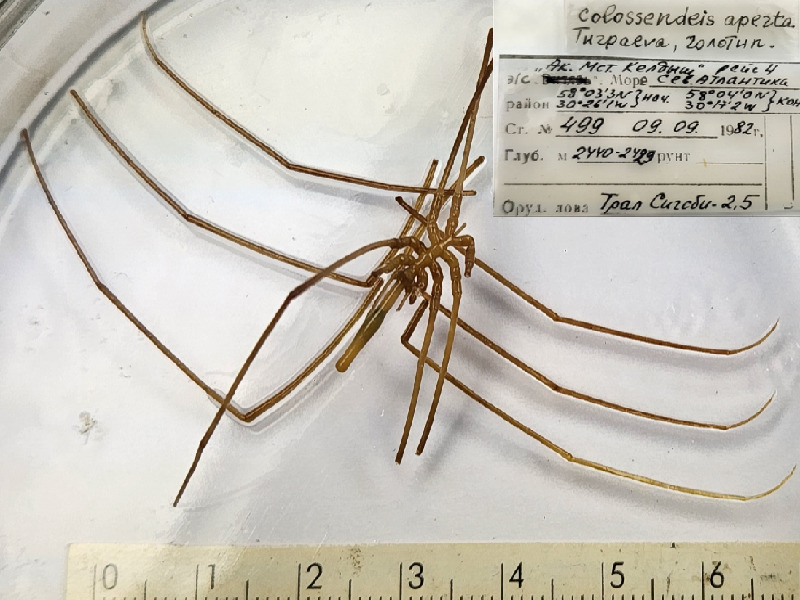
*Colossendeisaperta* (cat. INV0001288);

**Figure 4e.**
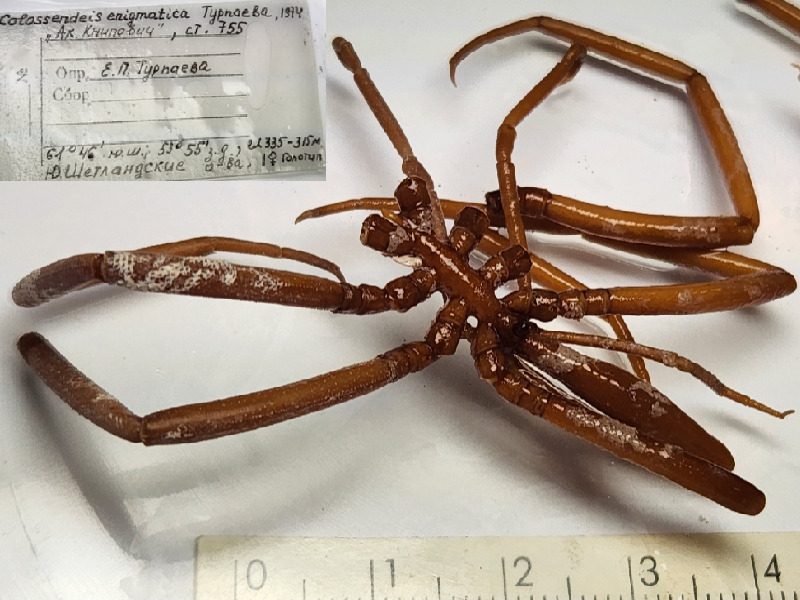
*Colossendeisenigmatica* (cat. INV0002343);

**Figure 4f.**
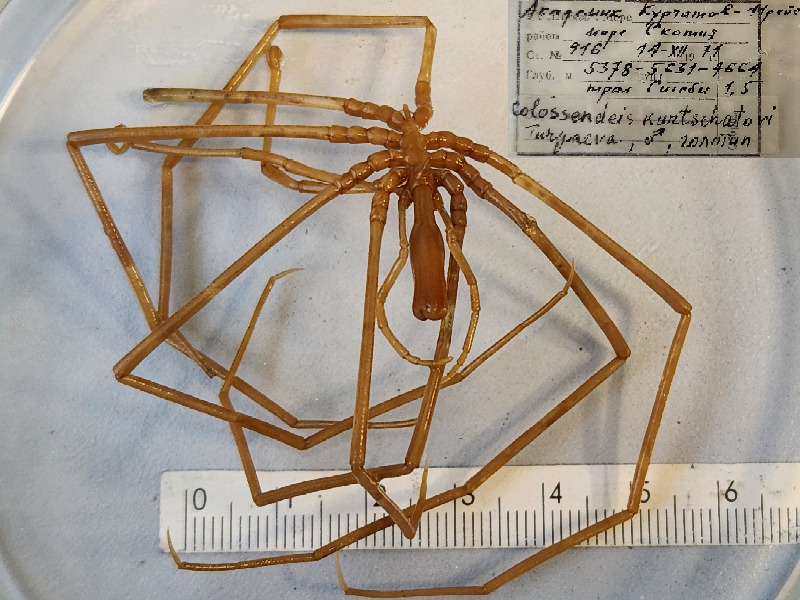
*Colossendeiskurtchatovi* (cat. INV0002351).

**Figure 5a.**
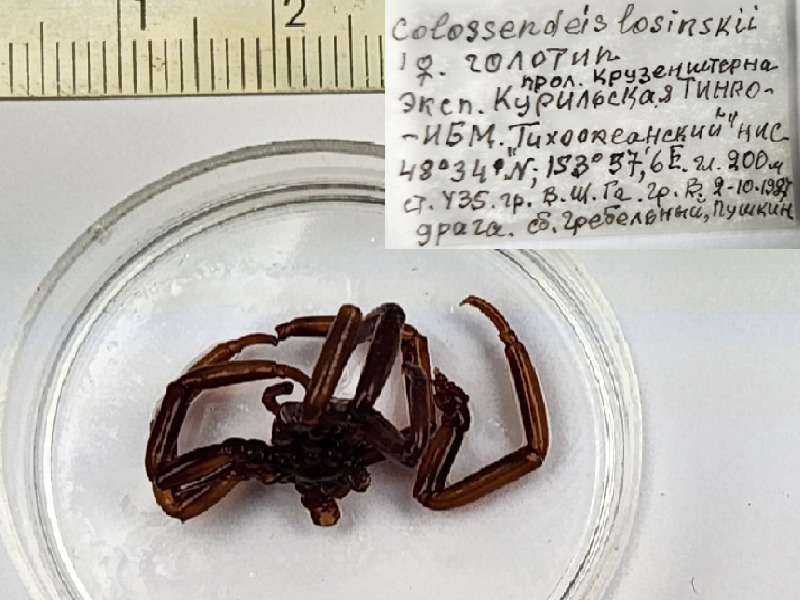
*Colossendeislosinskii* (cat. INV0001226);

**Figure 5b.**
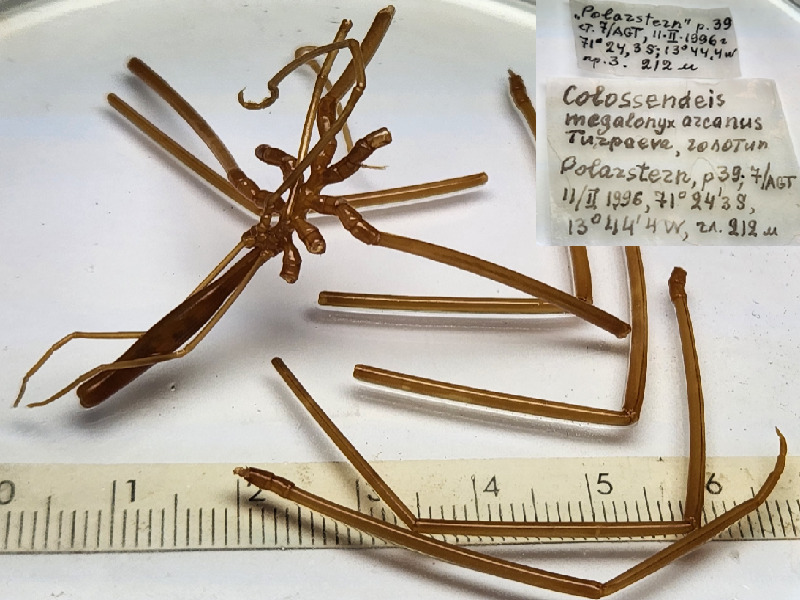
*Colossendeismegalonyxarcanus* (cat. INV0003247);

**Figure 5c.**
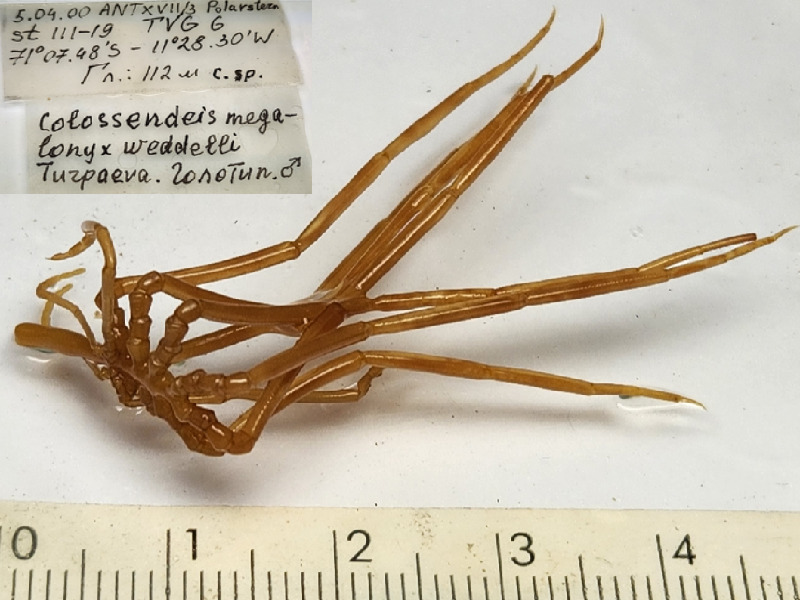
*Colossendeismegalonyxweddellensis* (cat. INV0003245);

**Figure 5d.**
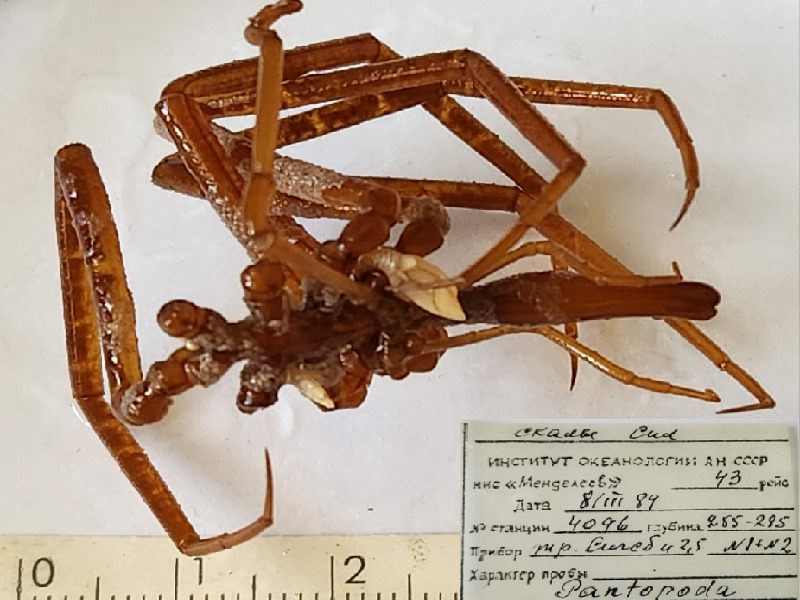
*Colossendeisperforata* (cat. INV0001475);

**Figure 5e.**
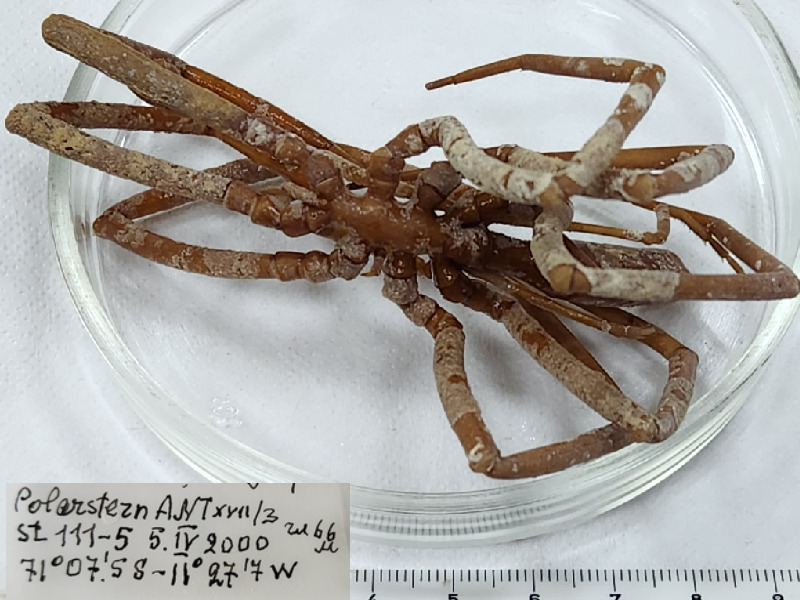
*Colossendeispotentis* (cat. INV0001872);

**Figure 5f.**
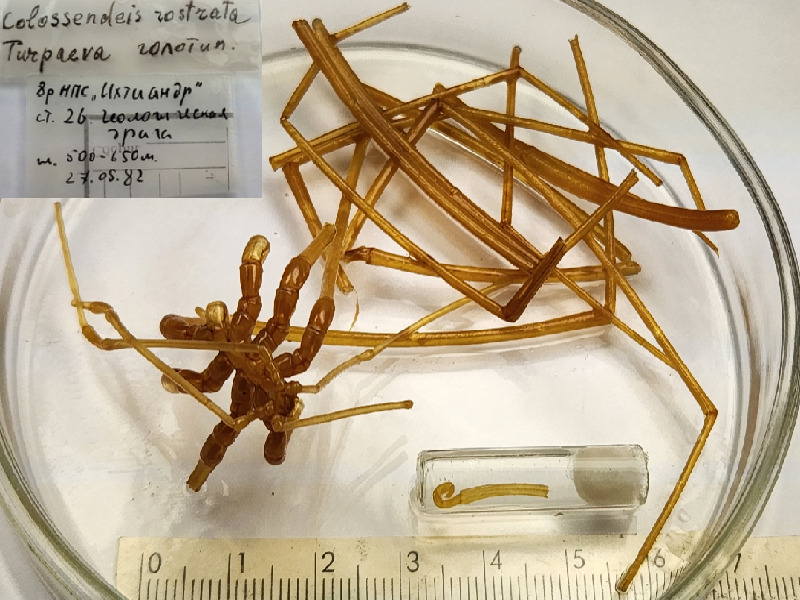
*Colossendeisrostrata* (cat. INV0001104).

**Figure 6a.**
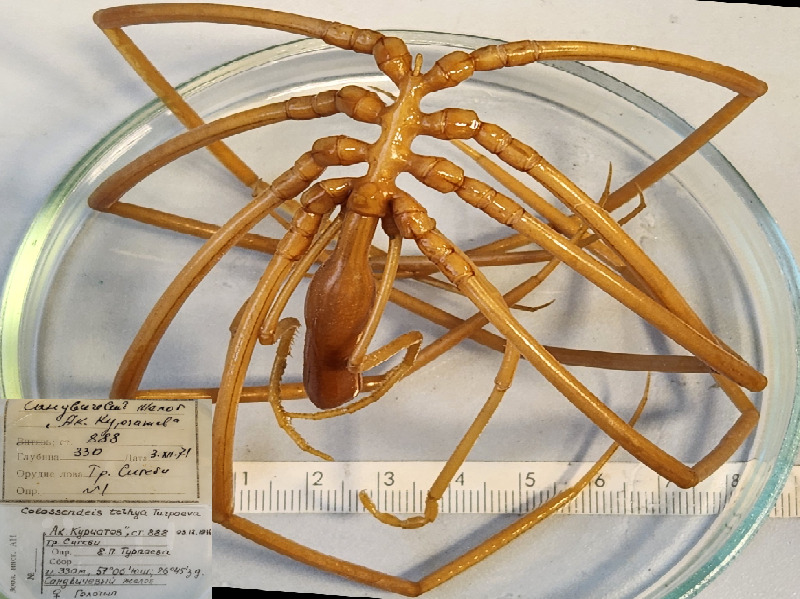
*Colossendeistethya* (cat. INV0002345);

**Figure 6b.**
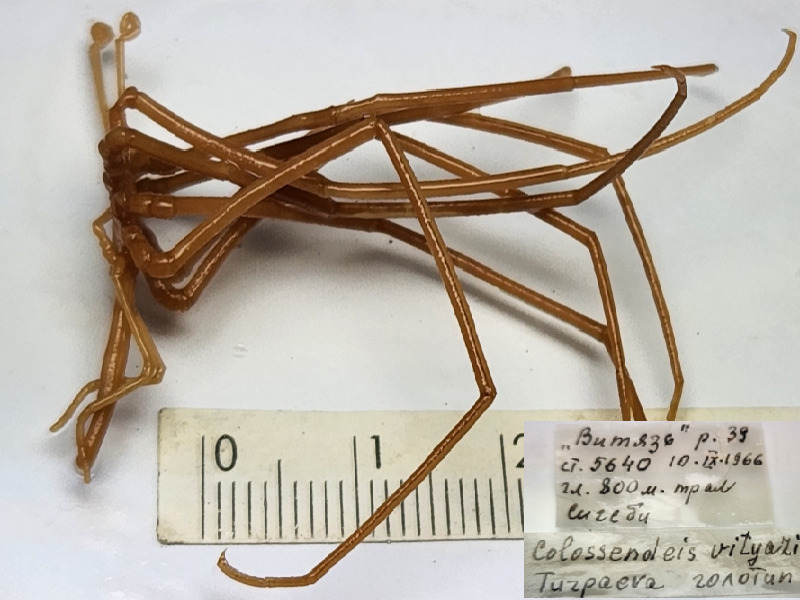
*Colossendeisvityazi* (cat. INV0002352);

**Figure 6c.**
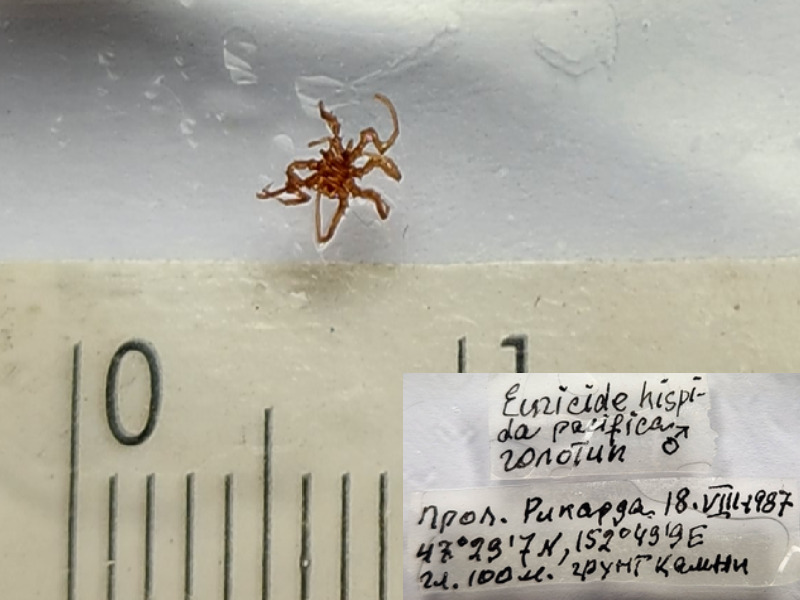
*Eurycydehispidaminor* (cat.INV0001758);

**Figure 6d.**
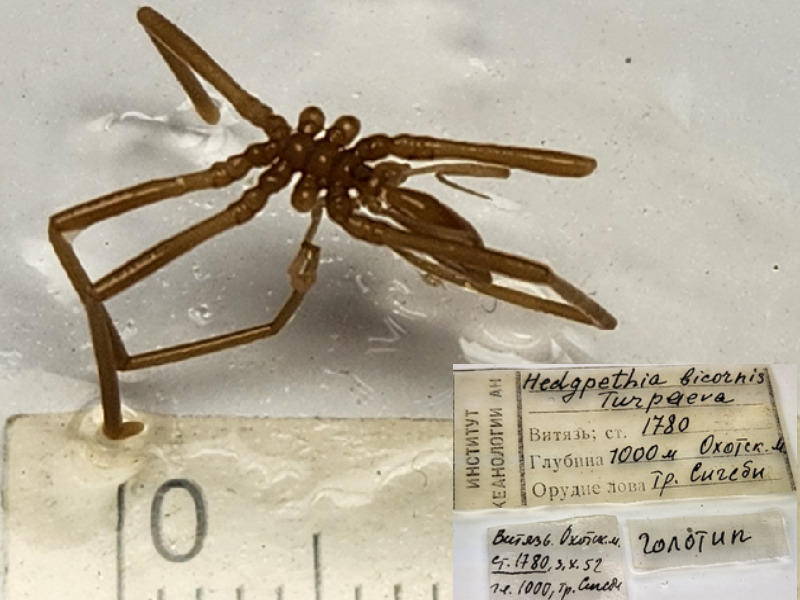
*Hedgpethiacalifornicabicornis* (cat. INV0002674);

**Figure 6e.**
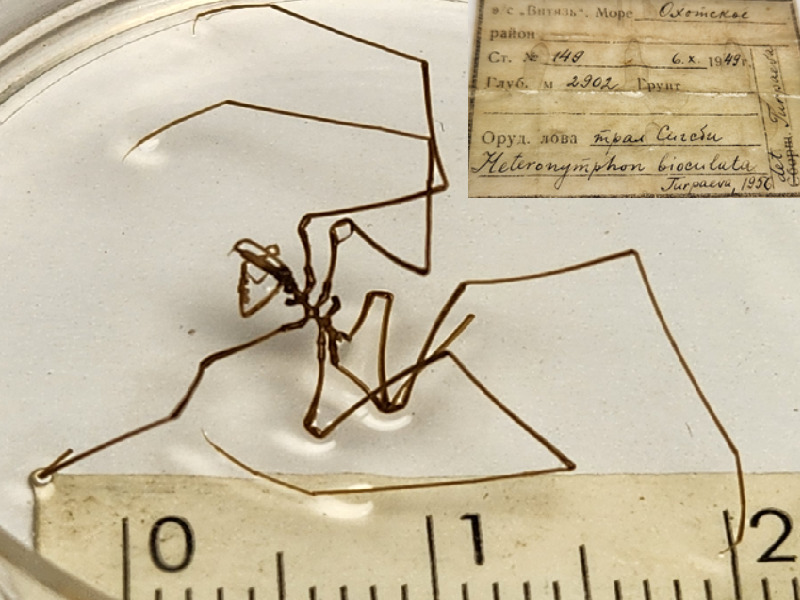
*Heteronymphonbioculatum* (cat. INV0003281);

**Figure 6f.**
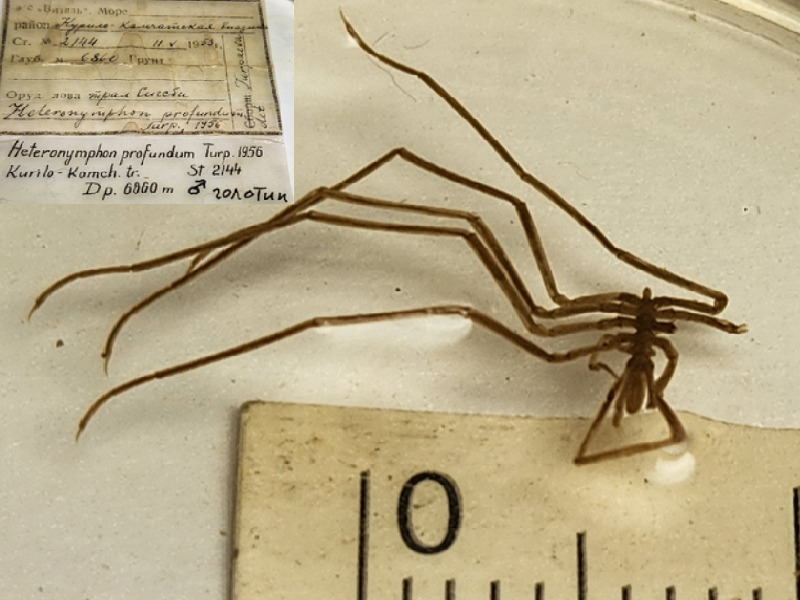
*Heteronymphonprofundum* (cat. INV0003280).

**Figure 7a.**
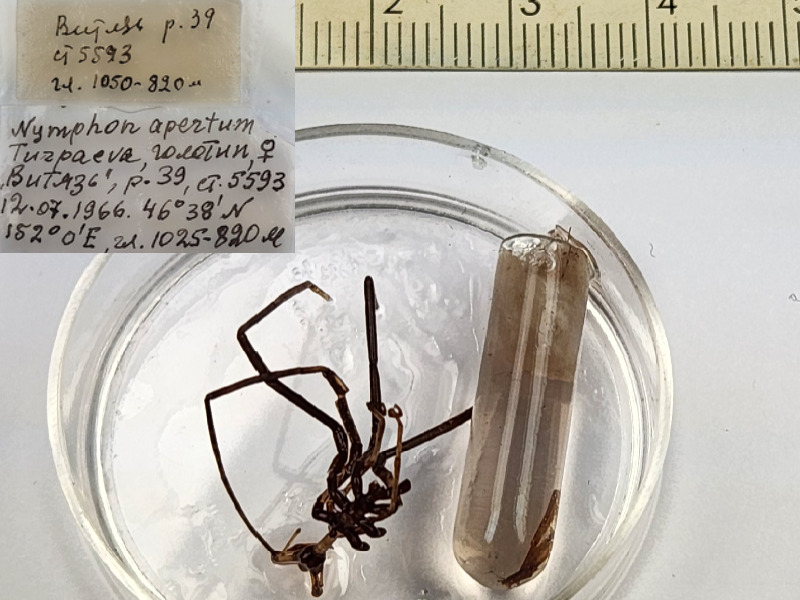
*Nymphonapertum* (cat. INV0003280);

**Figure 7b.**
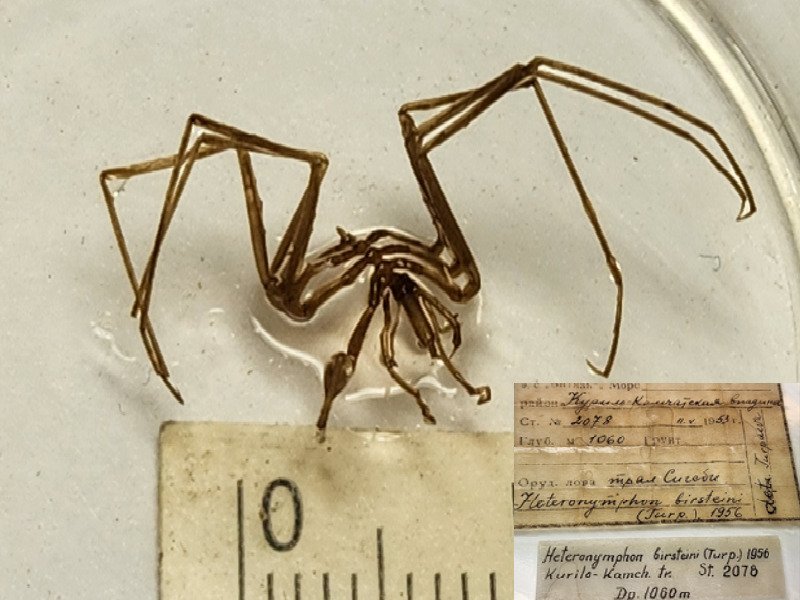
*Nymphonbirsteini* (cat. INV0003249);

**Figure 7c.**
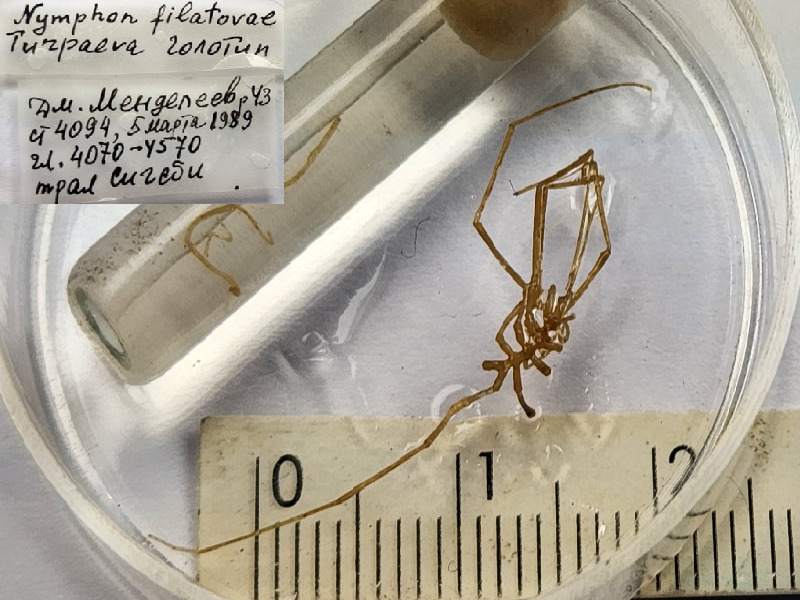
*Nymphonfilatovae* (cat. INV0000921);

**Figure 7d.**
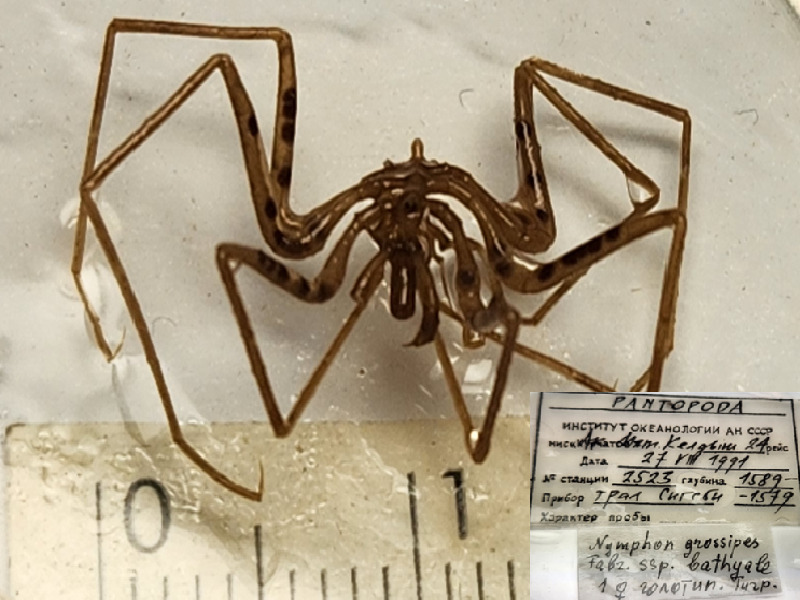
*Nymphongrossipesbathyale* (cat. INV0003275);

**Figure 7e.**
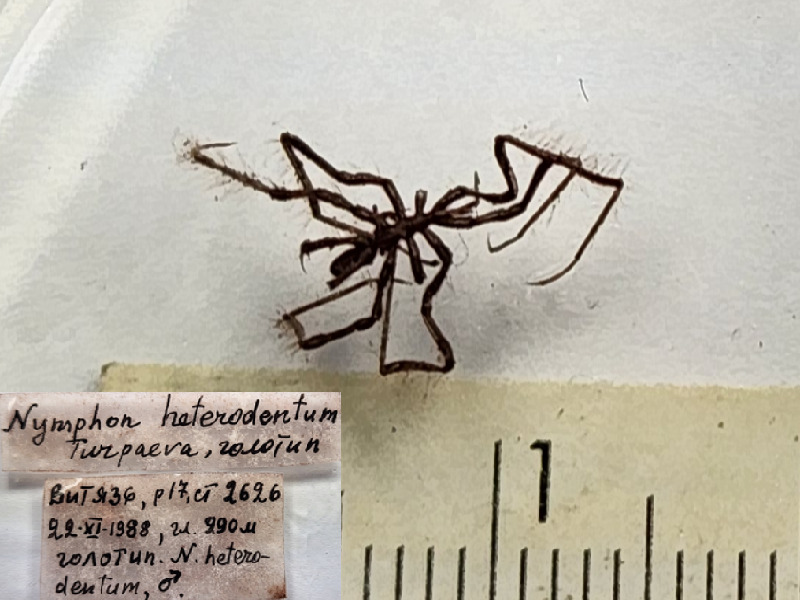
*Nymphonheterodentum* (cat. INV0000923);

**Figure 7f.**
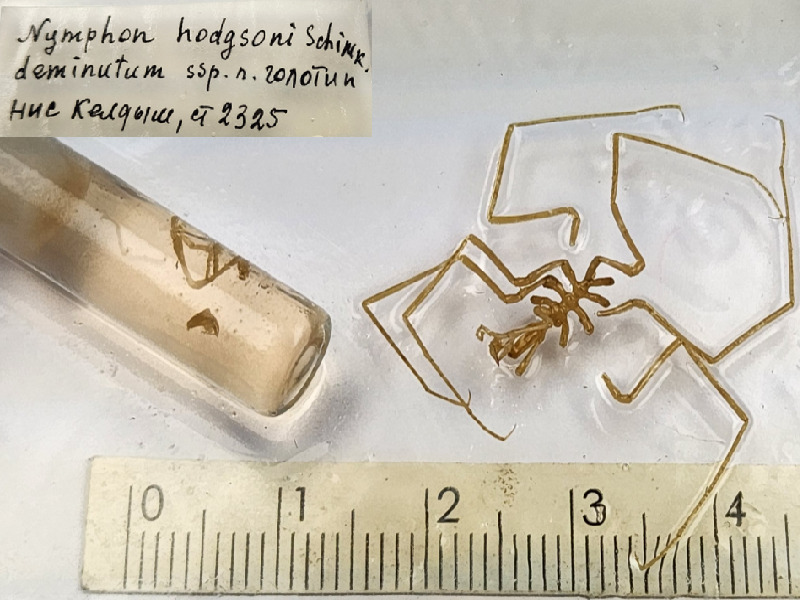
*Nymphonhodgsonidentimanum* (cat. INV0001344).

**Figure 8a.**
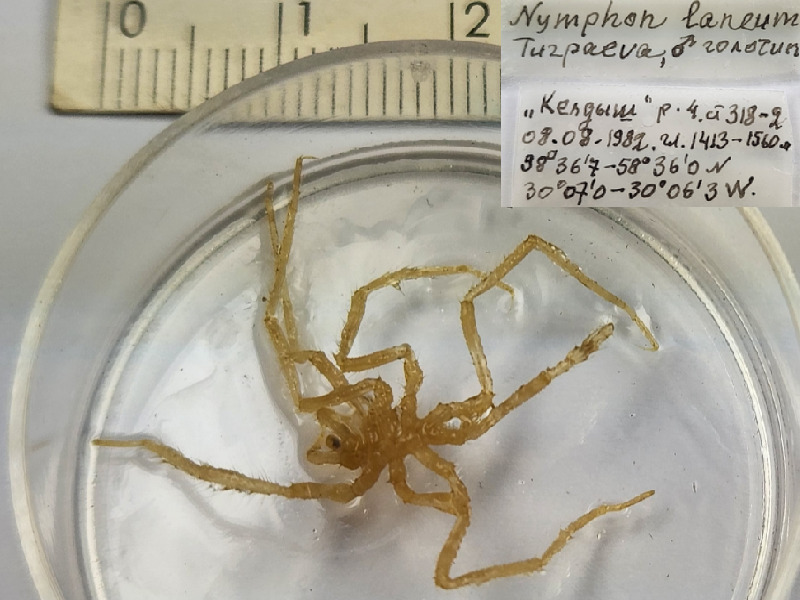
*Nymphonlaneum* (cat. INV0001286);

**Figure 8b.**
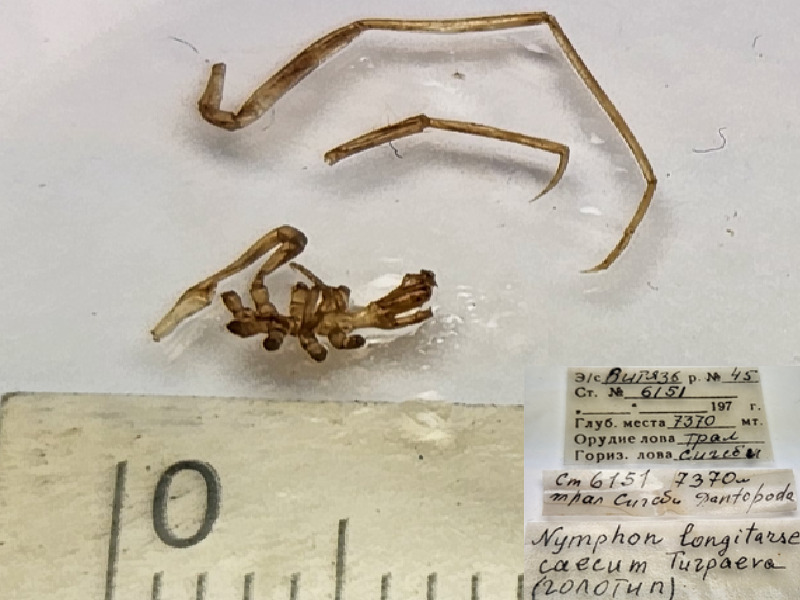
*Nymphonlongitarsecaecum* (cat. INV0002356);

**Figure 8c.**
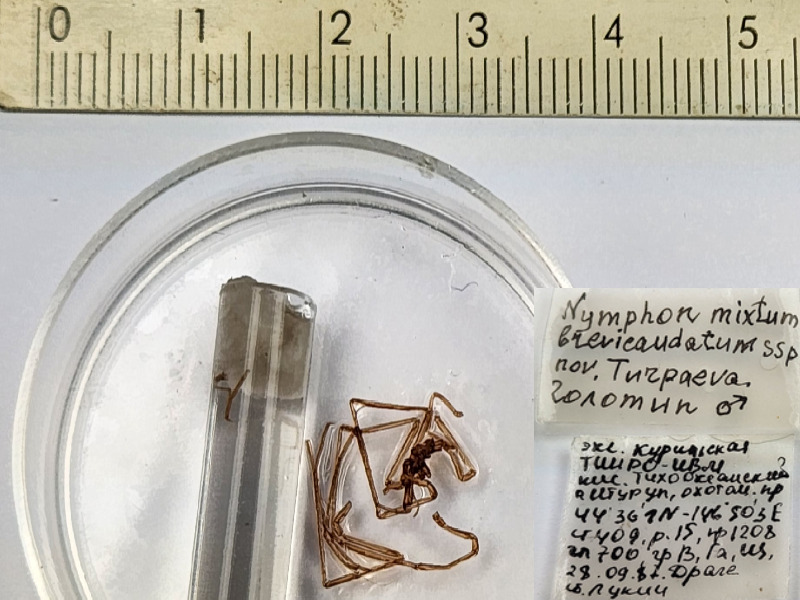
*Nymphonmixtumbrevicaudatum* (cat. INV0001233);

**Figure 8d.**
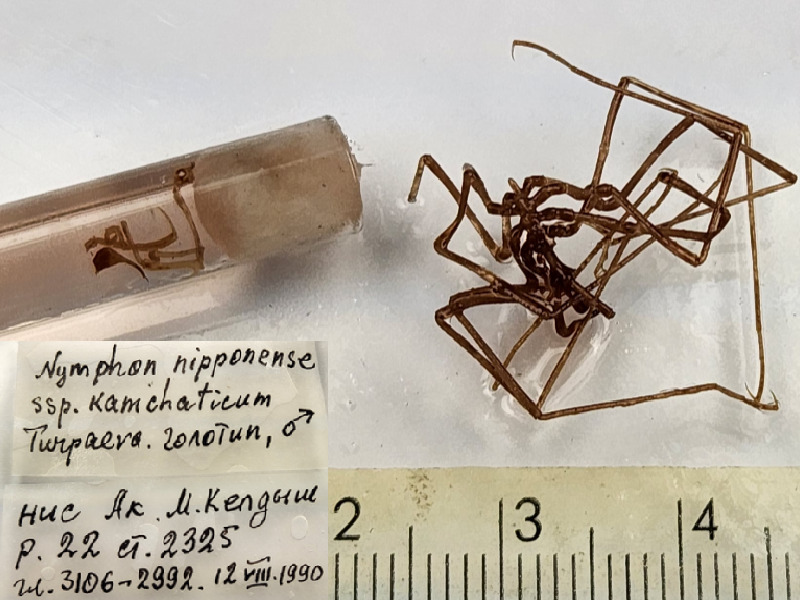
*Nymphonnipponensekamchaticum* (cat. INV0001324);

**Figure 8e.**
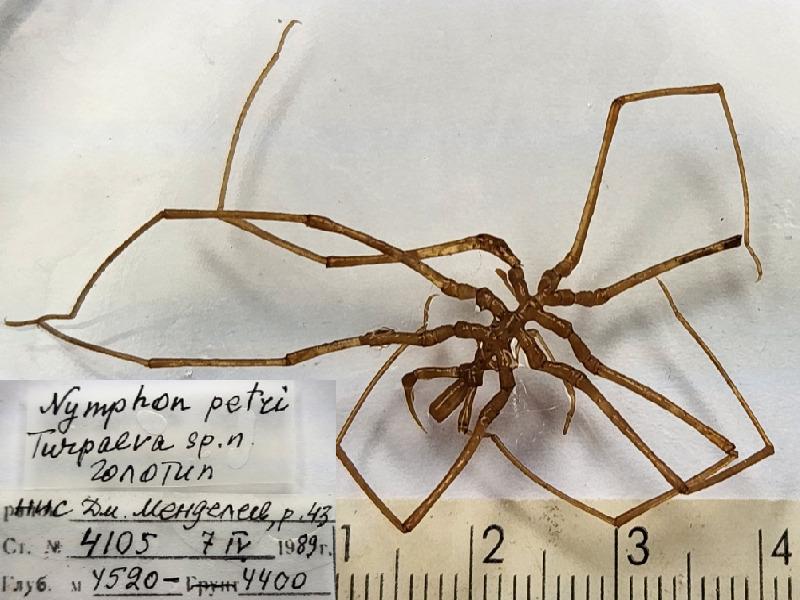
*Nymphonpetri* (cat. INV0000922);

**Figure 8f.**
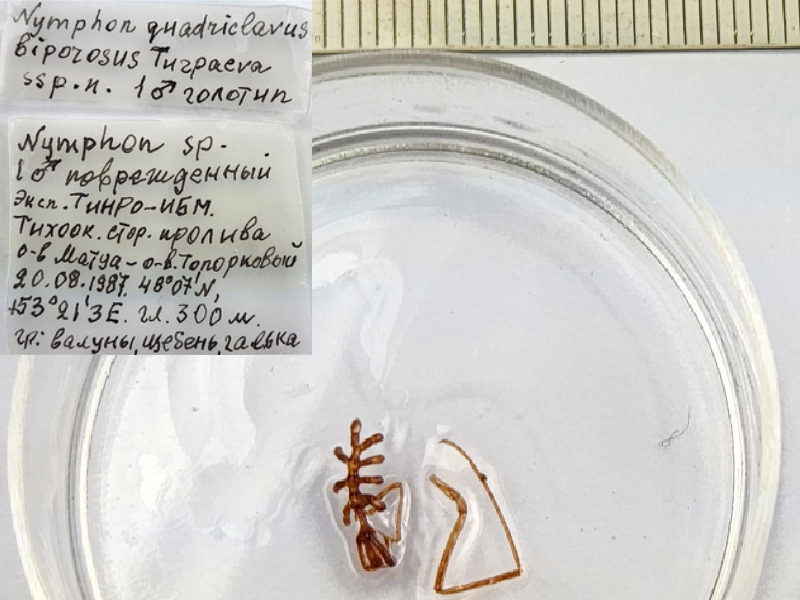
*Nymphonquadriclavusbiporosum* (cat. INV0000922).

**Figure 9a.**
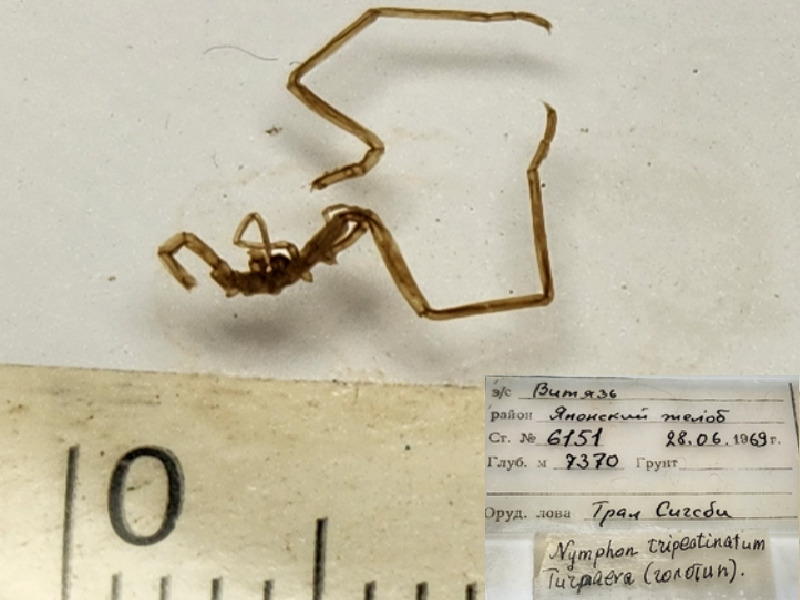
*Nymphontripectinatum* (cat. INV0002354);

**Figure 9b.**
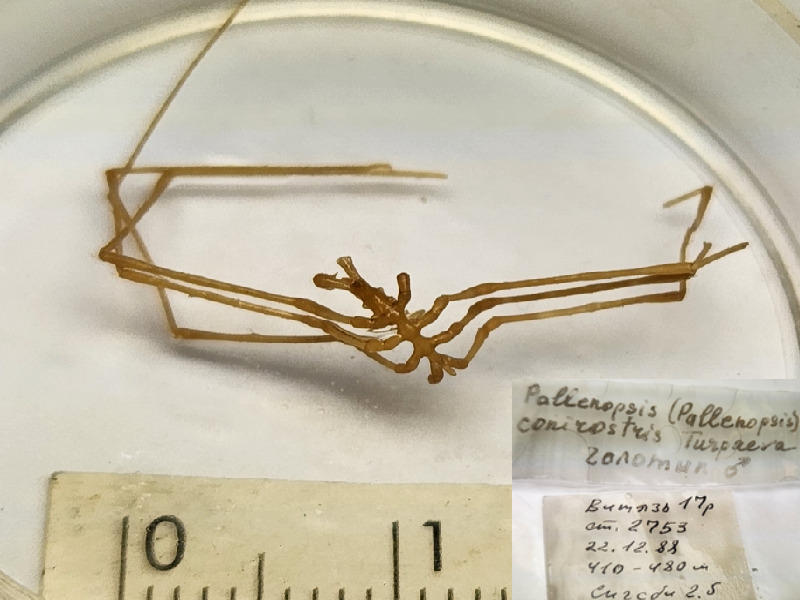
*Pallenopsisconirostris* (cat. INV0002360);

**Figure 9c.**
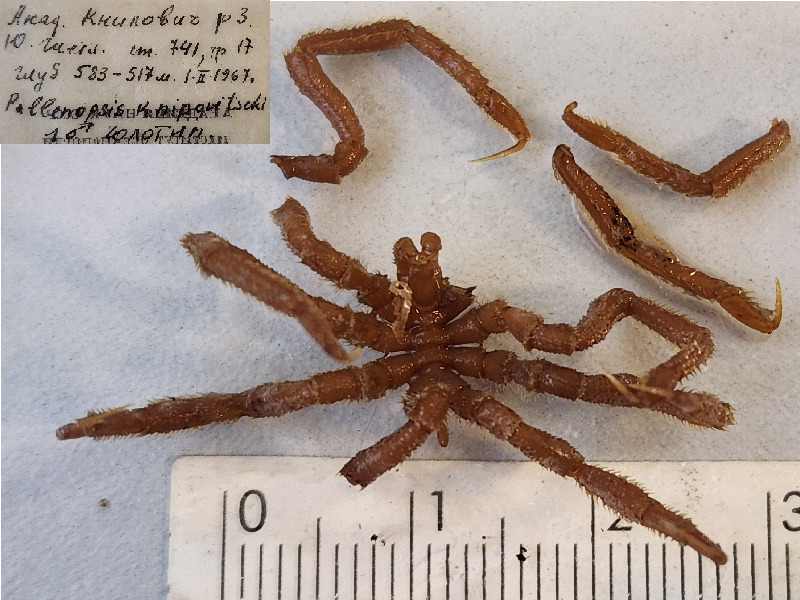
*Pallenopsisknipovichi* (cat. INV0002347);

**Figure 9d.**
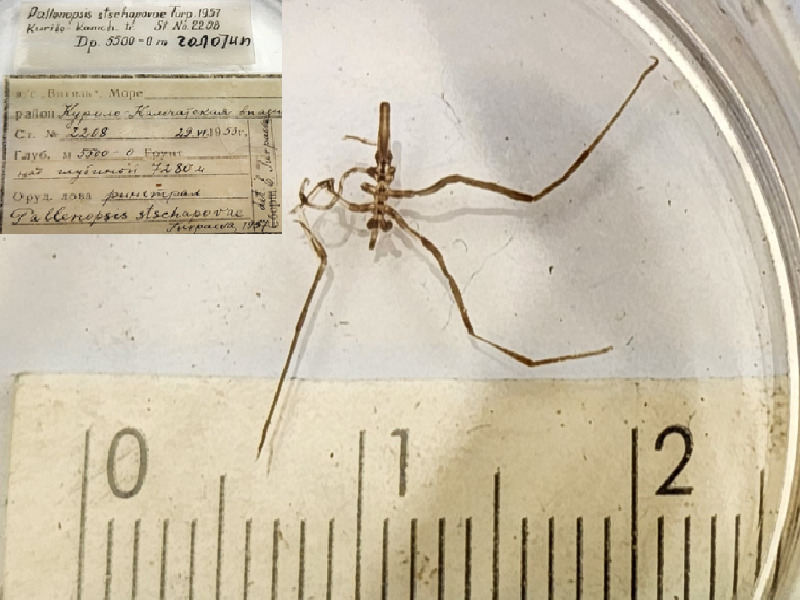
*Pallenopsisstschapovae* (cat. INV0001062);

**Figure 9e.**
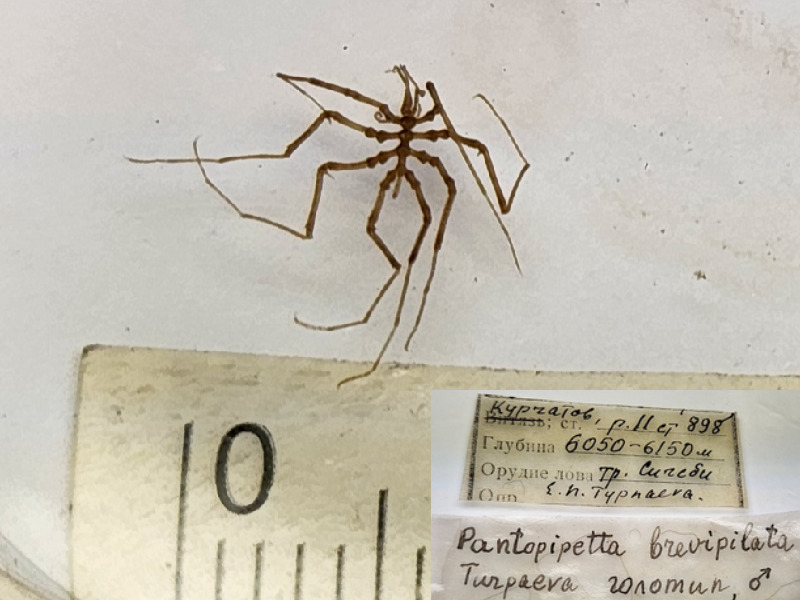
*Pantopipettabrevipilata* (cat. INV0002348);

**Figure 9f.**
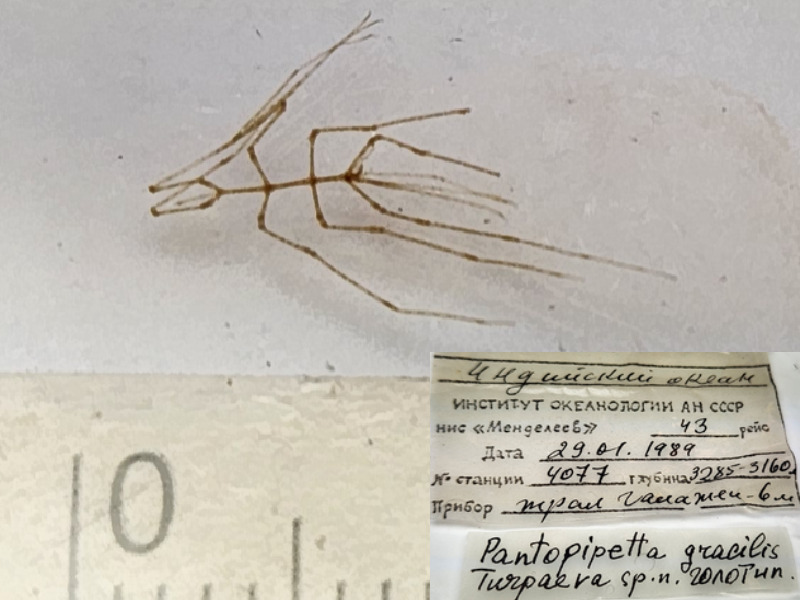
*Pantopipettagracilis* (cat. INV0002346).

**Figure 10a.**
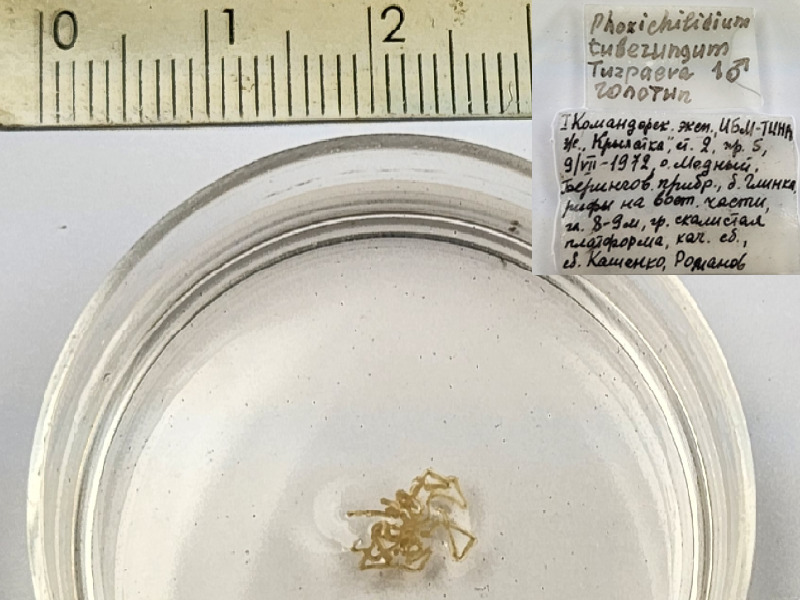
*Phoxichilidiumtuberungum* (cat. INV0001235);

**Figure 10b.**
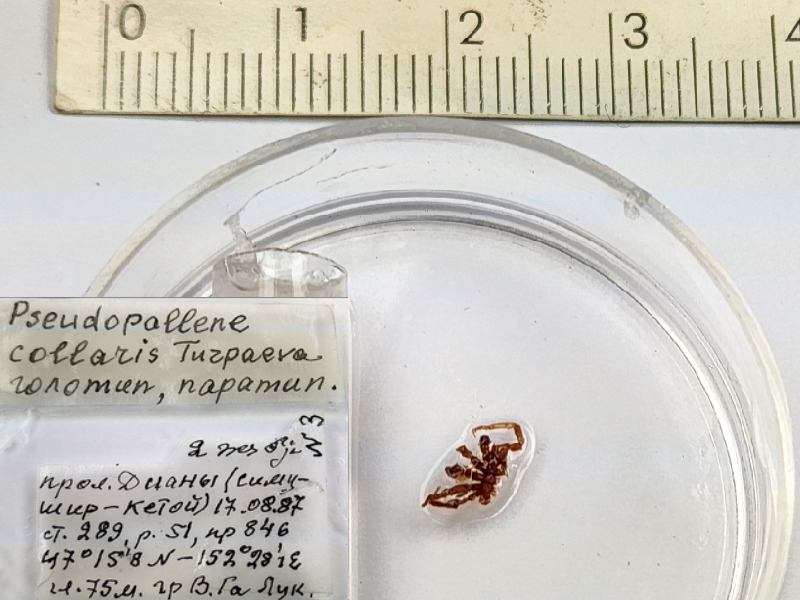
*Pseudopallenecollaris* (cat. INV0001238);

**Figure 10c.**
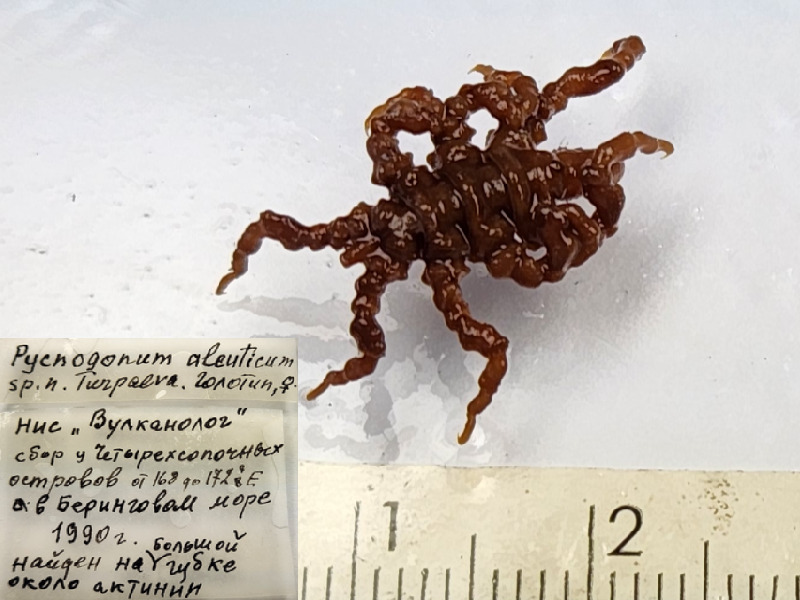
*Pycnogonumaleuticum* (cat. INV0001343);

**Figure 10d.**
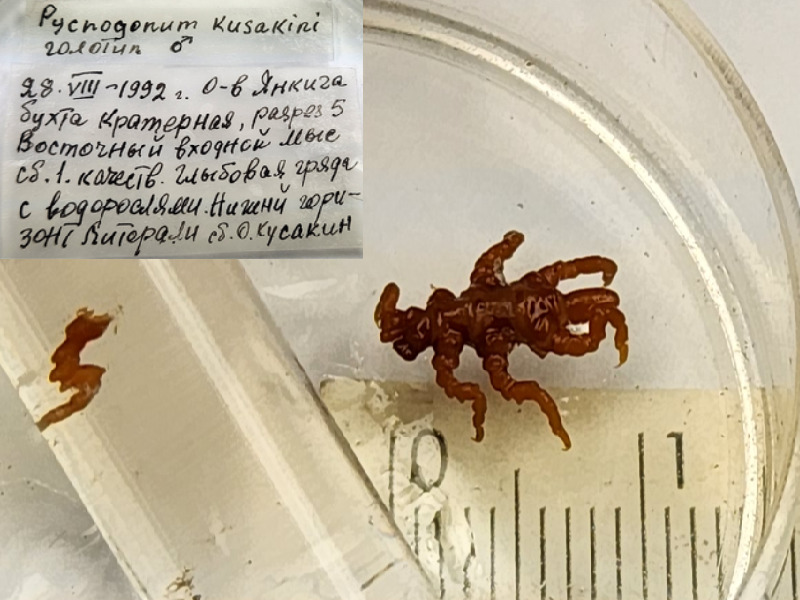
*Pycnogonumkussakini* (cat. INV0000974);

**Figure 10e.**
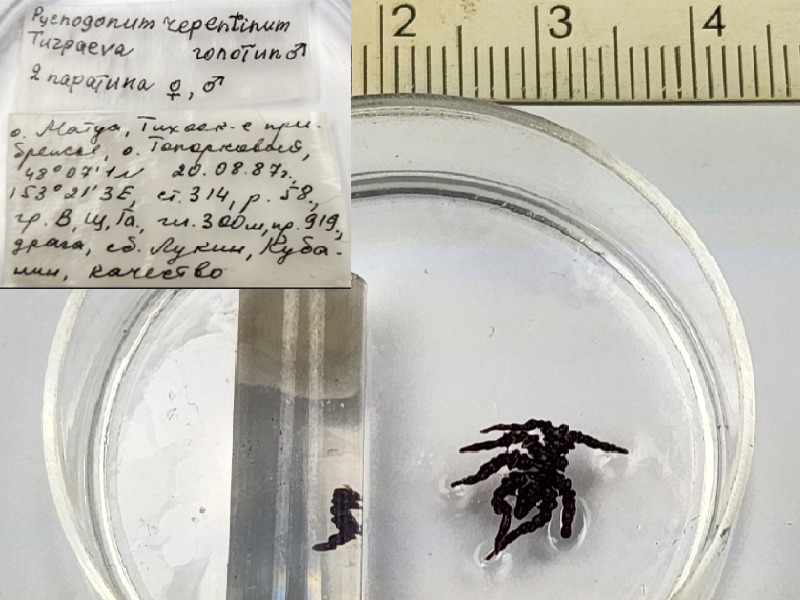
*Pycnogonumrepentinum* (cat. INV0001230);

**Figure 10f.**
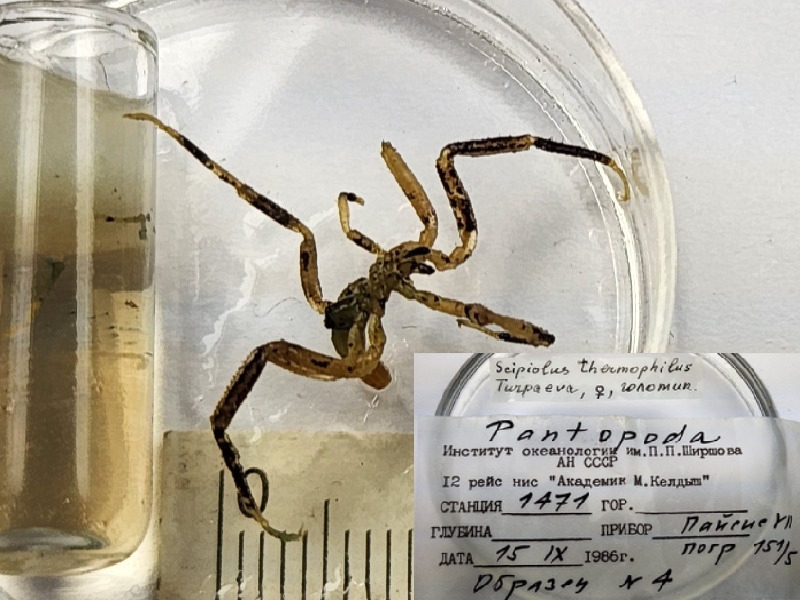
*Scipiolusthermophilus* (cat. INV0000925).

**Table 1.** Type specimens in the IORAS Pycnogonida collection. Number of paratypes are given in brackets after the catalogue number.

**No.**	**Scientific Name**	Reference	Figure	**Family**	**Type Status**	**Catalogue Number**
1	*Acheliaalaskensispacifica* Turpaeva, 2007	Turpaeva (2007)	Fig. 1a	Ammotheidae	Holotype	INV0001682
2	*Acheliaalaskensispacifica*			Ammotheidae	Paratypes	INV0001679 (2), INV0001680 (1), INV0001681 (2)
3	*Acheliaeuryfrontalis* Turpaeva, 2000	Turpaeva (2000)	Fig. 1b	Ammotheidae	Holotype	INV0000971
4	*Acheliaeuryfrontalis*			Ammotheidae	Paratype	INV0001480 (1)
5	*Acheliagrancapis* Turpaeva, 2007	Turpaeva (2007)	Fig. 1c	Ammotheidae	Holotype	INV0001690
6	*Acheliamicrosetosa* Turpaeva, 2007	Turpaeva (2007)	*Fig. 1d*	Ammotheidae	Holotype	INV0001688
7	*Acheliamicrosetosa*			Ammotheidae	Paratypes	INV0001689 (2)
8	*Acheliarostrata* Turpaeva, 2000	Turpaeva (2000)	Fig. 1e	Ammotheidae	Holotype	INV0000968
9	*Ammothellajaponica* Turpaeva, 1990 (currently accepted as *Cilunculusjaponicus* (Turpaeva, 1990)	Turpaeva (1990b)	Fig. 1f	Ammotheidae	Holotype	INV0000928
10	*Anoplodactylusgibbifemoris* Turpaeva, 1991	Turpaeva (1991a)	Fig. 2a	Phoxichilidiidae	Holotype	INV0002357
11	*Anoplodactylusgibbifemoris*			Phoxichilidiidae	Paratypes	INV0002358 (1), INV0002359 (1), INV0002599 (4)
12	*Anoplodactylusglobotuberculosus* Turpaeva, 2006	Turpaeva (2006)	Fig. 2b	Phoxichilidiidae	Holotype	INV0001244
13	*Anoplodactylustuberculosus* Turpaeva, 2006	Turpaeva (2006)	Fig. 2c	Phoxichilidiidae	Holotype	INV0001245
14	*Anoplodactylustuberculosus*			Phoxichilidiidae	Paratypes	INV0001246 (1), INV0001247 (1)
15	*Anoplodactylusviriosus* Turpaeva, 2006	Turpaeva (2006)	Fig. 2d	Phoxichilidiidae	Holotype	INV0001250
16	*Anoplodactylusviriosus*			Phoxichilidiidae	Paratypes	INV0001251 (1), INV0001252 (1)
17	*Ascorhynchusbirsteini* Turpaeva, 1971	Turpaeva (1971a)	Fig. 2e	Ascorhynchidae	Holotype	INV0002362
18	*Ascorhynchusbirsteini* Turpaeva, 1971			Ascorhynchidae	Paratype	INV0002597 (1)
19	*Ascorhynchusbucerus* Turpaeva, 1971	Turpaeva (1971a)	Fig. 2f	Ascorhynchidae	Syntypes	INV0000990 (2)
20	*Ascorhynchushedgpethi* Turpaeva, 1974	Turpaeva (1974)	Fig. 3a	Ascorhynchidae	Holotype	INV0000965
21	*Ascorhynchushippos* Turpaeva, 1994	Turpaeva (1994)	Fig. 3b	Ascorhynchidae	Holotype	INV0000793
22	*Ascorhynchushippos*			Ascorhynchidae	Paratype	INV0000792 (1)
23	*Ascorhynchuslevivani* Turpaeva, 1994	Turpaeva (1994)	Fig. 3c	Ascorhynchidae	Holotype	INV0002340
24	*Ascorhynchuslosinalosinskii* Turpaeva, 1971	Turpaeva (1971b)	Fig. 3d	Ascorhynchidae	Holotype	INV0002361
25	*Ascorhynchuslosinalosinskii*			Ascorhynchidae	Paratypes	INV0002598 (6)
26	*Ascorhynchusmariae* Turpaeva, 1971	Turpaeva (1971b)	Fig. 3e	Ascorhynchidae	Holotype	INV0002353
27	*Ascorhynchusmariae*			Ascorhynchidae	Paratypes	INV0002596 (5)
28	*Austrodecusvaldiviens* Turpaeva, 1990	Turpaeva (1990a)	Fig. 3f	Austrodecidae	Holotype	INV0000927
29	*Austropallenelukini* Turpaeva, 2002	Turpaeva (2002)	Fig. 4a	Callipallenidae	Holotype	INV0001228
30	*Cilunculuskunashiri* Turpaeva, 2007	Turpaeva (2007)	Fig. 4b	Ammotheidae	Holotype	INV0001757
31	*Cilunculusmisesetosus* Turpaeva, 2005	Turpaeva (2005)	Fig. 4c	Ammotheidae	Holotype	INV0000969
32	*Colossendeisaperta* Turpaeva, 2005	Turpaeva (2005)	Fig. 4d	Colossendeidae	Holotype	INV0001288
33	*Colossendeisaperta*			Colossendeidae	Paratypes	INV0001289 (2), INV0001290 (1)
34	*Colossendeisenigmatica* Turpaeva, 1974	Turpaeva (1974)	Fig. 4e	Colossendeidae	Holotype	INV0002343
35	*Colossendeisenigmatica* Turpaeva, 1974			Colossendeidae	Paratypes	INV0002344 (3)
36	*Colossendeiskurtchatovi* Turpaeva, 1993	Turpaeva (1993b)	Fig. 4f	Colossendeidae	Holotype	INV0002351
37	*Colossendeiskurtchatovi*			Colossendeidae	Paratypes	INV0003244 (1), INV0002350 (1)
38	*Colossendeislosinskii* Turpaeva, 2002	Turpaeva (2002)	Fig. 5a	Colossendeidae	Holotype	INV0001226
39	*Colossendeislosinskii*			Colossendeidae	Paratype	INV0001227 (1)
40	*Colossendeismegalonyxarcanus* (Turpaeva, 2008)	Turpaeva and Rajsky (2013)	Fig. 5b	Colossendeidae	Holotype	INV0003247
41	*Colossendeismegalonyxarcanus*			Colossendeidae	Paratypes	INV0003248 (6)
42	*Colossendeismegalonyxweddellensis* (Turpaeva, 2008)	Turpaeva and Rajsky (2013)	Fig. 5c	Colossendeidae	Holotype	INV0003245
43	*Colossendeismegalonyxweddellensis*			Colossendeidae	Paratypes	INV0003246 (2)
44	*Colossendeisperforata* Turpaeva, 1993	Turpaeva (1993a)	Fig. 5d	Colossendeidae	Holotype	INV0001475
45	*Colossendeispotentis* Turpaeva, 2008	Turpaeva (2008)	Fig. 5e	Colossendeidae	Holotype	INV0001872
46	*Colossendeisrostrata* Turpaeva, 1994	Turpaeva (1994)	Fig. 5f	Colossendeidae	Holotype	INV0001104
47	*Colossendeistethya* Turpaeva, 1974	Turpaeva (1974)	Fig. 6a	Colossendeidae	Holotype	INV0002345
48	*Colossendeisvityazi* Turpaeva, 1993	Turpaeva (1993b)	Fig. 6b	Colossendeidae	Holotype	INV0002352
49	*Colossendeisvityazi*			Colossendeidae	Paratype	INV0003242 (1)
50	*Eurycydehispidaminor* Turpaeva, 2007	Turpaeva (2007)	Fig. 6c	Ascorhynchidae	Holotype	INV0001758
51	*Eurycydehispidaminor*			Ascorhynchidae	Paratype	INV0001769 (1)
52	*Hedgpethiacalifornicabicornis* (Turpaeva, 1958)	Losina-Losinsky and Turpaeva (1958), Turpaeva (1973)	Fig. 6d	Colossendeidae	Holotype	INV0002674
53	*Hedgpethiacalifornicabicornis*			Colossendeidae	Paratypes	INV0002675 (3)
54	*Heteronymphonbioculatum* Turpaeva, 1956	Turpaeva (1956)	Fig. 6e	Nymphonidae	Holotype	INV0003281
55	*Heteronymphonbioculatum*			Nymphonidae	Paratype	INV0002244 (1)
56	*Heteronymphonprofundum* Turpaeva, 1956	Turpaeva (1956)	Fig. 6f	Nymphonidae	Holotype	INV0003280
57	*Nymphonapertum* Turpaeva, 2004	Turpaeva (2004)	Fig. 7a	Nymphonidae	Holotype	INV0001231
58	*Nymphonbirsteini* Turpaeva, 1955	Turpaeva (1955)	Fig. 7b	Nymphonidae	Holotype	INV0003249
59	*Nymphonfilatovae* Turpaeva, 1993	Turpaeva (1993a)	Fig. 7c	Nymphonidae	Holotype	INV0000921
60	*Nymphongrossipesbathyale* Turpaeva, 2005 (currently accepted as *Nymphongrossipes* (O. Fabricius, 1780) Fabricius 1780, Fabricius et al. 1794)	Turpaeva (2005)	Fig. 7d	Nymphonidae	Holotype	INV0003275
61	*Nymphongrossipesbathyale* (currently accepted as *Nymphongrossipes*)			Nymphonidae	Paratypes	INV0003276 (4), INV0003277 (8), INV0003278 (30), INV0003279 (2)
62	*Nymphonheterodentum* Turpaeva, 1991	Turpaeva (1991b)	Fig. 7e	Nymphonidae	Holotype	INV0000923
63	*Nymphonheterodentum*			Nymphonidae	Paratypes	INV0000924 (5)
64	*Nymphonhodgsonidentimanum* Turpaeva, 1994	Turpaeva (1994)	Fig. 7f	Nymphonidae	Holotype	INV0001344
65	*Nymphonhodgsonidentimanum*			Nymphonidae	Paratypes	INV0001345 (63), INV0001346 (2)
66	*Nymphonlaneum* Turpaeva, 2006	Turpaeva (2006)	Fig. 8a	Nymphonidae	Holotype	INV0001286
67	*Nymphonlaneum*			Nymphonidae	Paratypes	INV0001287 (39)
68	*Nymphonlongitarsecaecum* Turpaeva, 1971	Turpaeva (1971c)	Fig. 8b	Nymphonidae	Holotype	INV0002356
69	*Nymphonmixtumbrevicaudatum* Turpaeva, 2004 (currently accepted as *Nymphongrossipes*)	Turpaeva (2004)	Fig. 8c	Nymphonidae	Holotype	INV0001233
70	*Nymphonmixtumbrevicaudatum* (currently accepted as *Nymphongrossipes*)			Nymphonidae	Paratypes	INV0001234 (3)
71	*Nymphonnipponensekamchaticum* Turpaeva, 1994 (currently accepted as *Nymphonnipponense* Hedgpeth, 1949)	Turpaeva (1994)	Fig. 8d	Nymphonidae	Holotype	INV0001324
72	*Nymphonnipponensekamchaticum* (currently accepted as *Nymphonnipponense*)			Nymphonidae	Paratypes	INV0001059 (44), INV0001350 (45), INV0001351 (160), INV0001352 (5), INV0001353 (1)
73	*Nymphonpetri* Turpaeva, 1993	Turpaeva (1993a)	Fig. 8e	Nymphonidae	Holotype	INV0000922
74	*Nymphonquadriclavusbiporosum* Turpaeva, 2004	Turpaeva (2004)	Fig. 8f	Nymphonidae	Holotype	INV0001248
75	*Nymphonquadriclavusbiporosum*			Nymphonidae	Paratype	INV0001249 (1)
76	*Nymphontripectinatum* Turpaeva, 1971	Turpaeva (1971c)	Fig. 9a	Nymphonidae	Holotype	INV0002354
77	*Pallenopsisconirostris* Turpaeva, 1991	Turpaeva (1991b)	Fig. 9b	Pallenopsidae	Holotype	INV0002360
78	*Pallenopsisknipovichi* Turpaeva, 1974 (currently accepted as *Pallenopsismacronyx* Bouvier, 1911)	Turpaeva (1974)	Fig. 9c	Pallenopsidae	Holotype	INV0002347
79	*Pallenopsisknipovichi* (currently accepted as *Pallenopsismacronyx*)			Pallenopsidae	Paratypes	INV0002650 (10)
80	*Pallenopsislongiseta* Turpaeva, 1957 (currently accepted as *Bathypallenopsislongiseta* (Turpaeva, 1957)	Turpaeva (1957)		Pallenopsidae	Paratype	INV0001460 (1)
81	*Pallenopsisstschapovae* Turpaeva, 1957 (currently accepted as *Bathypallenopsistritonis* (Hoek, 1883)	Turpaeva (1957)	Fig. 9d	Pallenopsidae	Holotype	INV0001062
82	*Pantopipettabrevipilata* Turpaeva, 1990	*Turpaeva (1990a)*	*Fig. 9e*	Austrodecidae	Holotype	INV0002348
83	*Pantopipettagracilis* Turpaeva, 1993	Turpaeva (1993a)	Fig. 9f	Austrodecidae	Holotype	INV0002346
84	*Phoxichilidiumtuberungum* Turpaeva, 2006	Turpaeva (2006)	Fig. 10a	Phoxichilidiidae	Holotype	INV0001235
85	*Phoxichilidiumtuberungum*			Phoxichilidiidae	Paratype	INV0001236 (1)
86	*Pseudopallenecollaris* Turpaeva, 2002	Turpaeva (2002)	Fig. 10b	Callipallenidae	Holotype	INV0001238
87	*Pseudopallenecollaris*			Callipallenidae	Paratype	INV0001237 (1)
88	*Pycnogonumaleuticum* Turpaeva, 1994	Turpaeva (1994)	Fig. 10c	Pycnogonidae	Holotype	INV0001343
89	*Pycnogonumkussakini* Turpaeva, 2000	Turpaeva (2000)	Fig. 10d	Pycnogonidae	Holotype	INV0000974
90	*Pycnogonumkussakini*			Pycnogonidae	Paratype	INV0000973 (1)
91	*Pycnogonumrepentinum* Turpaeva, 2003	Turpaeva (2003)	Fig. 10e	Pycnogonidae	Holotype	INV0001229
92	*Pycnogonumrepentinum*			Pycnogonidae	Paratypes	INV0001230 (2)
93	*Scipiolusthermophilus* Turpaeva, 1988 (currently accepted as *Sericosuraverenae* (Child, 1987)	Turpaeva (1988)	Fig. 10f	Ammotheidae	Holotype	INV0000925

**Table 2.** Trawl catches localities with highest diversity of pycnogonid species.

**RV name, cruise number, station number**	**Number of species**	**Gear**	**Date**	**Locality**	**Depth**	**Latitude**	**Longitude**
RV Akademik Mstislav Keldysh, cruise 22, station 2325	18	Sigsbee trawl	12-08-1990	Pacific Ocean, Kamchatka SE slope	3106–2992 m	53.46167	160.98833
RV Dmitry Mendeleev, cruise 43, station 4096	14	Sigsbee trawl	08-03-1989	Scotia Sea, Elephant Island, Rocks of Strength	285–260 m	−60.83333	−55.66667
RV Polarstern, cruise ANTXVII/3, station 149-1	13	Agassiz trawl	24-04-2000	Bransfield Strait	911–909 m	−62.5	−56.93
RV Polarstern, cruise ANT-XIII/3, station 39/01	12	bottom trawl	05-02-1996	Weddell Sea	462–481 m	−71.05167	−11.425
RV Polarstern, cruise ANTXVII/3, station 65-1	12	bottom trawl	31-03-2000	Weddell Sea	615–648 m	−71.29333	−13.8
RV Akademik Kurchatov, cruise 11, station 882	10	Sigsbee trawl	02-12-1971	Atlantic Ocean, Sandwich Trench	1687–1837 m	−57.15	−26.65
RV Akademik Knipovich, cruise 3, station 755	9	bottom trawl	01-02-1967	Bransfield Strait	335–315 m	−61.775	−53.92
RV Polarstern, cruise ANT-XIII/3, station 39/29	9	benthopelagic trawl	28-02-1996	Weddell Sea	504–529 m	−71.525	−12.425
RV Polarstern, cruise ANTXVII/3, station 102-1	9	bottom trawl	03-04-2000	Weddell Sea	260–310 m	−71.22	−12.465
RV Vityaz, cruise 52, station 6669	9	Sigsbee trawl	22-06-1972	Northeast Pacific Ocean	425 m	39.98	−142.32833
RV Akademik Mstislav Keldysh, cruise 54, station 4960	8	Sigsbee trawl	12-09-2007	Kara Sea	123 m	71.41267	64.86983
RV Polarstern, cruise ANTXVII/3, station 136-1	8	bottom trawl	10-04-2000	Weddell Sea	271–251 m	−70.83667	−13.59
RV Polarstern, cruise ANTXVII/3, station 165-1	8	Agassiz trawl	28-04-2000	Bransfield Strait	621–618 m	−63.01333	−59.115
RV Polarstern, cruise ARK XI/1, station 36/083a	8	Agassiz trawl	07-09-1995	Laptev Sea	311 m	77.94833	113.57833
RV Professor Shtokman, cruise 81, station 5	8	small trawl	08-09-2006	Kara Sea, Stepovoy Bay	43–32 m	72.55757	55.4548
RV Akademik Kurchatov, cruise 11, station 888-1	8	Sigsbee trawl	03-12-1971	Atlantic Ocean, South Sandwich Islands	318 m	−57.1	−26.73333
RV Vityaz, cruise 39, station 5594	8	Sigsbee trawl	12-07-1966	Northwest Pacific Ocean, Kurile Islands	1440– 1540 m	46.63333	152.05

**Table 3.** Ultra-abyssal specimens in the IORAS collection.

**Scientific Name**	**Catalogue Number**	**Type Status**	**Depth**	**Locality**	**Collecting Event**	**Date**
*Hedgpethiachitinosa* (Hilton, 1943)	INV0002604		6410–6757 m	Aleutian Trench	RV Vityaz cruise 20 station 3340	01-06-1955
Pantopoda indet.	INV0001446		6890–6770 m	Izu-Bonin Trench	RV Vityaz cruise 57 station 7404	09-05-1975
*Nymphon* sp.	INV0002254		7370 m	Japan Trench	RV Vityaz cruise 45 station 6151	28-06-1969
*Nymphon* sp.	INV0002255		7370 m	Japan Trench	RV Vityaz cruise 45 station 6151	28-06-1969
*Nymphonlongitarse* Krøyer, 1844	INV0002252		7370 m	Japan Trench	RV Vityaz cruise 45 station 6151	28-06-1969
*Nymphonlongitarsecaecum* Turpaeva, 1971	INV0002253		7370 m	Japan Trench	RV Vityaz cruise 45 station 6151	28-06-1969
*Nymphonlongitarsecaecum*	INV0002356	Holotype	7370 m	Japan Trench	RV Vityaz cruise 45 station 6151	28-06-1969
*Nymphontripectinatum* Turpaeva, 1971	INV0002354	Holotype	7370 m	Japan Trench	RV Vityaz cruise 45 station 6151	28-06-1969
*Heteronymphonprofundum* Turpaeva, 1956	INV0002806		6156–6207 m	Japan Trench	RV Vityaz cruise 19 station 3214	24-10-1954
*Heteronymphonprofundum*	INV0002808		6380.0 m	Japan Trench	RV Vityaz cruise 24 station 3593	22-05-1957
*Nymphonprocerum* Hoek, 1881	INV0002873		6156–6117 m	Kurile-Kamchatka Trench	RV Vityaz cruise 39 station 5633	06-09-1966
*Pantopipettalongituberculata* (Turpaeva, 1955)	INV0002800		6156–6117 m	Kurile-Kamchatka Trench	RV Vityaz cruise 39 station 5633	06-09-1966
*Pantopipettalongituberculata*	INV0002799		6710–6675 m	Kurile-Kamchatka Trench	RV Vityaz cruise 39 station 5617	06-09-1966
*Pallenopsisstschapovae* Turpaeva, 1957	INV0001062	Holotype	7280 m	Kurile-Kamchatka Trench	RV Vityaz cruise 14 station 2208	22-06-1953
*Bathypallenopsiscalcanea* (Stephensen, 1933)	INV0002628		8185–8400 m	Kurile-Kamchatka Trench	RV Vityaz cruise 39 station 5612	27-07-1966
*Heteronymphonprofundum* Turpaeva, 1956	INV0003280	Holotype	6860 m	Kurile-Kamchatka Trench	RV Vityaz cruise 14 station 2144	01-06-1953
*Achelia* sp.	INV0001448		10700–10730 m	Mariana Trench	RV Vityaz cruise 57 station 7359	23-04-1975
*Endeis* sp.	INV0005251		10700–10730 m	Mariana Trench	RV Vityaz cruise 57 station 7359	23-04-1975
*Ascorhynchusbirsteini* Turpaeva, 1971	INV0002362	Holotype	6040 m	Peru Trench	RV Akademik Kurchatov cruise 4 station 296	02-11-1968
*Ascorhynchusbirsteini*	INV0002597	Paratype	6040 m	Peru Trench	RV Akademik Kurchatov cruise 4 station 296	02-11-1968
*Pantopipettabrevipilata* Turpaeva, 1990	INV0002348	Holotype	6150–6052 m	South Sandwich Trench	RV Akademik Kurchatov cruise 11 station 898	05-12-1971
Pantopoda indet.	INV0001449		6330–6320 m	Volkano Trench	RV Vityaz cruise 57 station 7391	05-05-1975

**Table 4.** Number of collection lots by genera.

**Genus**	**Nr**	**Genus**	**Nr**
*Nymphon* Fabricius, 1794	781	*Endeis* Philippi, 1843	6
*Colossendeis* Jarzynsky, 1870	302	*Austroraptus* Hodgson, 1907	4
*Achelia* Hodge, 1864	129	*Chaetonymphon* Sars, 1888	3
*Boreonymphon* Sars, 1888	87	*Parapallene* Carpenter, 1892	3
*Pallenopsis* Wilson, 1881	62	*Tanystylum* Miers, 1879	3
*Ascorhynchus* Sars, 1877	50	*Ecleipsothremma* Fry & Hedgpeth, 1969	2
*Ammothea* Leach, 1814	41	*Pycnosomia* Losina-Losinsky, 1961	2
*Anoplodactylus* Wilson, 1878	37	*Rhynchothorax* Costa, 1861	2
*Phoxichilidium* Milne Edwards, 1840	31	*Scipiolus* Loman, 1908	2
*Anisopes* Turpaeva, 1998	30	*Pentapycnon* Bouvier, 1910	2
*Heteronymphon* Gordon, 1932	26	*Bathypallenopsis* Stock, 1975	1
*Cordylochele* Sars, 1888	23	*Decachela* Hilton, 1939	1
*Austropallene* Hodgson, 1915	19	*Seguapallene* Pushkin, 1975	1
*Hedgpethia* Turpaeva, 1973	18	*Athernopycnon* Fry & Hedgpeth, 1969	1
*Pantopipetta* Stock, 1963	18	*Biammothea* Pushkin, 1993	1
*Eurycyde* Schiödte, 1857	17	*Callipallene* Flynn, 1929	1
*Pseudopallene* Wilson, 1878	12	*Dodecolopoda* Calman & Gordon, 1933	1
*Austrodecus* Hodgson, 1907	11	*Leionymphon* Möbius, 1902	1
*Cilunculus* Loman, 1908	11	*Oropallene* Schimkewitsch, 1930	1
*Pycnogonum* Brünnich, 1764	11	*Paranymphon* Caullery, 1896	1
*Lecythorhynchus* Böhm, 1879	9	*Phoxiphilyra* Stock, 1974	1
*Decolopoda* Eights, 1835	7	*Rhopalorhynchus* Wood-Mason, 1873	1
*Pentanymphon* Hodgson, 1904	7	*Sexanymphon* Hedgpeth & Fry, 1964	1
